# Whole Blood Gene Expression Profiles to Assess Pathogenesis and Disease Severity in Infants with Respiratory Syncytial Virus Infection

**DOI:** 10.1371/journal.pmed.1001549

**Published:** 2013-11-12

**Authors:** Asuncion Mejias, Blerta Dimo, Nicolas M. Suarez, Carla Garcia, M. Carmen Suarez-Arrabal, Tuomas Jartti, Derek Blankenship, Alejandro Jordan-Villegas, Monica I. Ardura, Zhaohui Xu, Jacques Banchereau, Damien Chaussabel, Octavio Ramilo

**Affiliations:** 1 Division of Pediatric Infectious Disease, The Research Institute at Nationwide Children's Hospital, The Ohio State University College of Medicine, Columbus, Ohio, United States of America; 2 Center for Vaccines and Immunity, The Research Institute at Nationwide Children's Hospital, The Ohio State University College of Medicine, Columbus, Ohio, United States of America; 3 Division of Pediatric Infectious Diseases, University of Texas Southwestern Medical Center, Dallas, Texas, United States of America; 4 Department of Pediatrics, Turku University Hospital, Turku, Finland; 5 Department of Statistics, Baylor Research Institute, Dallas, Texas, United States of America; 6 Baylor Institute for Immunology Research, Baylor Research Institute, Dallas, Texas, United States of America; 7 Benaroya Research Institute, Seattle, Washington, United States of America; University of Liverpool, United Kingdom

## Abstract

**Background:**

Respiratory syncytial virus (RSV) is the leading cause of viral lower respiratory tract infection (LRTI) and hospitalization in infants. Mostly because of the incomplete understanding of the disease pathogenesis, there is no licensed vaccine, and treatment remains symptomatic. We analyzed whole blood transcriptional profiles to characterize the global host immune response to acute RSV LRTI in infants, to characterize its specificity compared with influenza and human rhinovirus (HRV) LRTI, and to identify biomarkers that can objectively assess RSV disease severity.

**Methods and Findings:**

This was a prospective observational study over six respiratory seasons including a cohort of infants hospitalized with RSV (*n* = 135), HRV (*n* = 30), and influenza (*n* = 16) LRTI, and healthy age- and sex-matched controls (*n* = 39). A specific RSV transcriptional profile was identified in whole blood (training cohort, *n* = 45 infants; Dallas, Texas, US) and validated in three different cohorts (test cohort, *n* = 46, Dallas, Texas, US; validation cohort A, *n* = 16, Turku, Finland; validation cohort B, *n* = 28, Columbus, Ohio, US) with high sensitivity (94% [95% CI 87%–98%]) and specificity (98% [95% CI 88%–99%]). It classified infants with RSV LRTI versus HRV or influenza LRTI with 95% accuracy. The immune dysregulation induced by RSV (overexpression of neutrophil, inflammation, and interferon genes, and suppression of T and B cell genes) persisted beyond the acute disease, and immune dysregulation was greatly impaired in younger infants (<6 mo). We identified a genomic score that significantly correlated with outcomes of care including a clinical disease severity score and, more importantly, length of hospitalization and duration of supplemental O_2_.

**Conclusions:**

Blood RNA profiles of infants with RSV LRTI allow specific diagnosis, better understanding of disease pathogenesis, and assessment of disease severity. This study opens new avenues for biomarker discovery and identification of potential therapeutic or preventive targets, and demonstrates that large microarray datasets can be translated into a biologically meaningful context and applied to the clinical setting.

*Please see later in the article for the Editors' Summary*

## Abstract 2

In this study, Mejias and colleagues found that specific blood RNA profiles of infants with RSV LRTI allowed for specific diagnosis, better understanding of disease pathogenesis, and better assessment of disease severity.

*Please see later in the article for the Editors' Summary*

## Editors' Summary

**Background:**

Lower respiratory tract infections (LRTIs)—bacterial and viral infections of the lungs and airways (the tubes that take oxygen-rich air to the lungs)—are major causes of illness and death in children worldwide. Pneumonia (infection of the lungs) alone is responsible for 14% of all child deaths. The leading cause of viral LTRIs in children is respiratory syncytial virus (RSV), which is readily transmitted from person to person by direct contact with nasal fluids or airborne droplets. Almost all children have an RSV infection before their second birthday, but most have only minor symptoms similar to those of a common cold and are cared for at home. Unfortunately, some children develop more serious conditions when they become infected with RSV, such as pneumonia or bronchiolitis (swelling and mucus build-up in the bronchioles, the smallest air passages in the lungs). These children have to be admitted to the hospital for supportive care—there is no specific treatment for RSV infection—such as the provision of supplemental oxygen.

**Why Was This Study Done?:**

The lack of a treatment (and of a vaccine) for RSV is largely due to our incomplete understanding of the cellular events and reactions, including the host immune response, that occur during the development of an RSV infection (disease pathogenesis). Moreover, based on physical examination and available diagnostic tools, it is impossible to predict which children infected with RSV will develop a serious condition that requires hospitalization and which ones can be safely nursed at home. Here, the researchers use microarrays to analyze the global host response to acute RSV LTRI in infants, to define gene expression patterns that are specific to RSV infection rather than infection with other common respiratory viruses, and to identify biomarkers that indicate the severity of RSV infection. “Microarray” analysis allows researchers to examine gene expression patterns (“RNA transcriptional profiles”) in, for example, whole blood; a biomarker is a molecule whose level in bodily fluids or tissues indicates how a disease might develop and helps with patient classification.

**What Did the Researchers Do and Find?:**

The researchers compared the RNA transcriptional profile in whole blood taken from children less than two years old hospitalized with RSV, human rhinovirus, or influenza virus infection (rhinovirus and influenza are two additional viral causes of LRTI), and from healthy infants. Using “statistical group comparisons,” they identified more than 2,000 transcripts that were differentially expressed in blood from 45 infants with RSV infection and from 14 healthy matched controls. Genes related to interferon function (interferons are released by host cells in response to the presence of disease-causing organisms) and neutrophil function (neutrophils are immune system cells that, like interferons, are involved in the innate immune response, the body's first line of defense against infection) were among the most overexpressed genes in infants infected with RSV. Genes regulating T and B cells (components of the adaptive immune response, the body's second-line of defense against infection) were among the most underexpressed genes. This specific transcriptional profile, which was validated in three additional groups of infants, accurately distinguished between infants infected with RSV and those infected with human rhinovirus or influenza virus. Finally, a “molecular distance to health” score (a numerical score that quantifies the transcriptional perturbation associated with an illness) was correlated with the clinical disease severity score of the study participants, with how long they needed supplemental oxygen, and with their duration of hospitalization.

**What Do These Findings Mean?:**

These findings suggest that it might be possible to use whole blood RNA transcriptional profiles to distinguish between infants infected with RSV and those with other viruses that commonly cause LRTI. Moreover, if these findings can be replicated in more patients (including non-hospitalized children), gene expression profiling might provide a strategy for triaging patients with RSV infections when they first present to an emergency department and for monitoring clinical changes during the course of the infection, particularly given the development of molecular tools that might soon enable the “real time” acquisition of transcriptional profiles in the clinical setting. Finally, although certain aspects of the study design limit the accuracy and generalizability of the study's findings, these data provide new insights into the pathogenesis of RSV infection and open new avenues for the discovery of biomarkers for RSV infection and for the identification of therapeutic and preventative targets.

**Additional Information:**

Please access these websites via the online version of this summary at http://dx.doi.org/10.1371/journal.pmed.1001549.

- This study is further discussed in a *PLOS Medicine* Perspective by Peter Openshaw
- The US Centers for Disease Control and Prevention provides information about RSV infection
- The US National Heart, Lung, and Blood Institute provides information about the respiratory system and about RSV infections
- The UK National Health Service Choices website provides information about bronchiolitis
- The British Lung Foundation also provides information on RSV and on bronchiolitis
- MedlinePlus provides links to other resources about RSV infections and about pneumonia (in English and Spanish); the MedlinePlus encyclopedia has a page on bronchiolitis (in English and Spanish)
- PATH is an international non-profit organization investigating new RSV vaccines

## Introduction

Lower respiratory tract infections (LRTIs) are associated with significant morbidity and mortality in children worldwide [1]. The implementation of vaccination with conjugate vaccines against the most common causes of bacterial LRTI, *Haemophilus influenzae* type b and *Streptococcus pneumoniae*, has clearly shown a protective effect in children [2],[3]. On the other hand, respiratory viruses account for a large proportion of LRTIs in children, greater than previously estimated [4],[5]. Respiratory syncytial virus (RSV) is the most common cause of viral LRTI leading to hospitalization in young children worldwide [6]–[8]. Despite the disease burden, there is no available vaccine, and treatment remains symptomatic. In addition, in the clinical setting it is impossible to predict, based on the physical examination and available diagnostic tools, which patients will progress to worse disease requiring hospitalization and which patients can be discharged home. Hence, there is a clear need to better understand the immune response to RSV and how it relates to disease pathogenesis, progression, and severity.

Through analyzing whole blood RNA transcriptional profiles, the major goals of this study were (1) to characterize the global host response to acute RSV LRTI in infants, (2) to define the specific profile of RSV infection compared with other common respiratory viruses including human rhinovirus (HRV) and influenza, and (3) to identify specific biomarkers that objectively assess RSV disease severity.

## Methods

### Ethics Statement

Our study complied with the guidelines of the Declaration of Helsinki. As such, the institutional review boards of University of Texas Southwestern Medical Center (#0802-447), Baylor Research Institute (#002-141), Turku University Hospital (#21.11.2006-492), and Nationwide Children's Hospital (#10-00028) approved the study. Written informed consent was obtained from legal guardians.

### Patient Characteristics

This was a prospective cohort study involving a convenience sample of children <2 y old hospitalized at Children's Medical Center (Dallas, Texas, US), Turku University Hospital (Turku, Finland), or Nationwide Children's Hospital (Columbus, Ohio, US) with RSV (*n* = 135), HRV (*n* = 30), or influenza A (*n* = 16) LRTI during six respiratory seasons (2006–2011). Samples from 39 healthy age-, race-, and sex-matched controls, and 1-mo follow-up samples from 21 children with RSV LRTI were also obtained (Table S1). Race (African American, White, Asian, Other) and ethnicity (Hispanic and non-Hispanic) were defined according to US National Institutes of Health criteria, and were captured by the research team via direct interview with caregivers. In addition to gender and age, race/ethnicity information was gathered to control for the influence of basic demographic characteristics between patients and controls when performing the analyses. RSV, influenza, and HRV infections were diagnosed by hospital standard methods (rapid antigen, direct fluorescent antibody, or PCR) [9].

Monday through Friday, patients were identified using the daily virology laboratory report. Those with confirmed RSV, influenza, or HRV infection underwent screening. We excluded children with documented bacterial co-infections (including bacteremia, urinary tract infection, meningitis, acute gastroenteritis, or any bacterial pathogen isolated from a sterile site) or viral co-infections. We also excluded children with congenital heart disease, chronic lung disease, immunodeficiency, prematurity (<36 wk), and systemic steroid treatment within 2 wk before presentation. Patients fulfilling inclusion criteria were enrolled, and blood samples for microarray analyses were collected at a median time of (interquartile range [IQR]) 48 (24–72) h after admission. Control samples were obtained from healthy children undergoing elective surgery not involving the respiratory tract, or at routine outpatient visits. For the healthy control group, a clinical questionnaire was used, and those children with co-morbidities, use of systemic steroids, or presence of any illness within 2 wk prior to enrollment were excluded. Lastly, to exclude viral co-infections, respiratory samples were tested by viral culture or PCR in 94% of patients and controls. Overall, our patient recruitment rates varied from 70% to 80% and 50% to 60% for patients with viral LRTI and healthy matched controls, respectively. The most frequent reasons for lack of participation reported by parents included lack of parental interest or no parental availability to sign the consent. In addition to the patients that did not participate, as mentioned above, we excluded from the analysis those already enrolled but who were found to have a bacterial or viral co-infection (∼20%) and those in whom the quantity or quality of RNA was insufficient. Despite these limitations, patients were enrolled in the study with various degrees of disease severity, over different respiratory seasons, and from different geographical locations, and are most likely a good representation of the patients that are cared for with RSV LRTI in hospitals.

### Study Design

**Figure 1:**
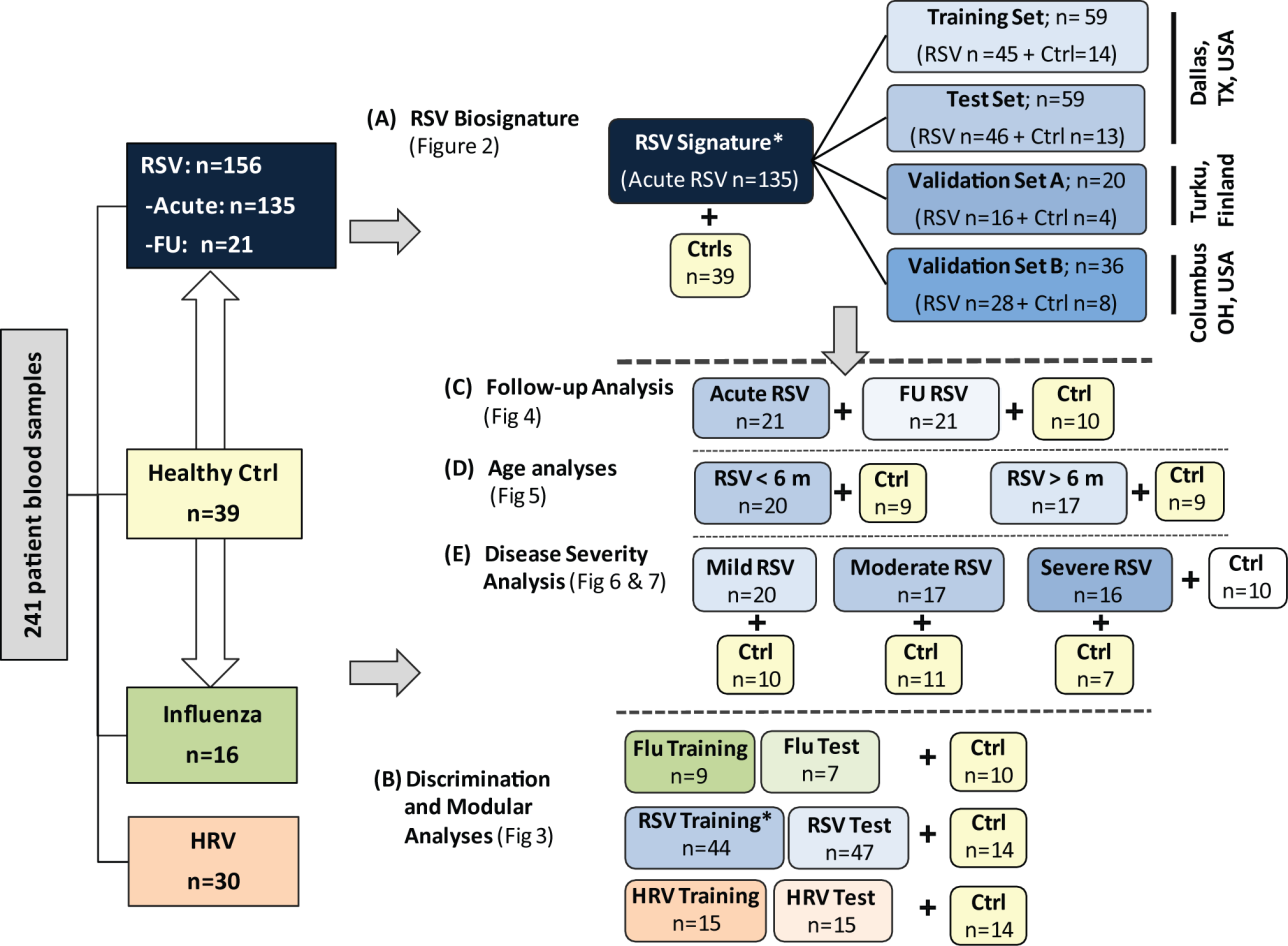
Flow diagram of study patients. Patient allocation and analyses performed throughout the study are depicted in Figure 1: RSV signature analysis (A), discrimination and modular analyses (B), follow-up analysis (C), age analyses (D), and disease severity analysis (E). Patients included in the different sub-analyses were matched for age, gender, and race/ethnicity with controls. In addition, for the age analyses (D), children greater or younger than 6 mo were matched for disease severity. Asterisk indicates that RSV patients used for viral discrimination analysis (B) were previously used in the RSV signature analysis (A). Ctrl, control; FU, follow-up.

For analysis purposes patients were classified as follows. (1) To define and validate the systemic host response to RSV, patients were divided in four groups: training and test sets enrolled at Children's Medical Center (Dallas, Texas), a second validation set (validation A) enrolled at Turku University Hospital (Turku, Finland), and a third validation set (validation B) enrolled at Nationwide Children's Hospital (Columbus, Ohio). (2) To define the specificity of the RSV biosignature, RSV transcriptional profiles were compared with those from children with HRV and influenza LRTI. (3) To characterize the differences in host responses to RSV according to age, transcriptional profiles were compared between infants <6 mo old versus those 6–24 mo of age. (4) To define how RSV disease severity was reflected at the transcriptional level, we compared transcriptional profiles from children with various degrees of disease severity based on a clinical disease severity score (CDSS) assessed at the time of sample collection [10],[11]. This clinical score was modified from the score originally described by Tal et al. [12] and comprises five parameters (respiratory rate, auscultation, transcutaneous O_2_ saturation, retractions, and level of activity, mainly assessed by feeding difficulty). Each parameter ranks from 0 (normal) to 3 (most abnormal). Using this score, patients were classified as having mild (0–5), moderate (6–10), or severe (11–15) RSV LRTI. The clinicians that collected the clinical information and the bioinformaticians that analyzed the data were blinded to the transcriptional and clinical data, respectively. The number of patients included in each analysis is displayed in Figure 1.

### Sample Collection

Blood samples (1–3 ml) were collected in Tempus tubes (Applied Biosystems) and stored at −20°C. Whole blood RNA was processed and hybridized into Illumina Human WG-6 v3 (for training, test, and validation A cohorts; 49,576 probes) or Human HT-12 v4 (for validation B cohort; 47,323 probes) beadchips and scanned on an Illumina Beadstation 500 system [13],[14]. The two Illumina beadchips shared 39,426 probes, with >95% probe compatibility. The data are deposited in the NCBI Gene Expression Omnibus (GEO accession number: GSE38900).

### Microarray Data and Statistical Analysis

**Figure 2:**
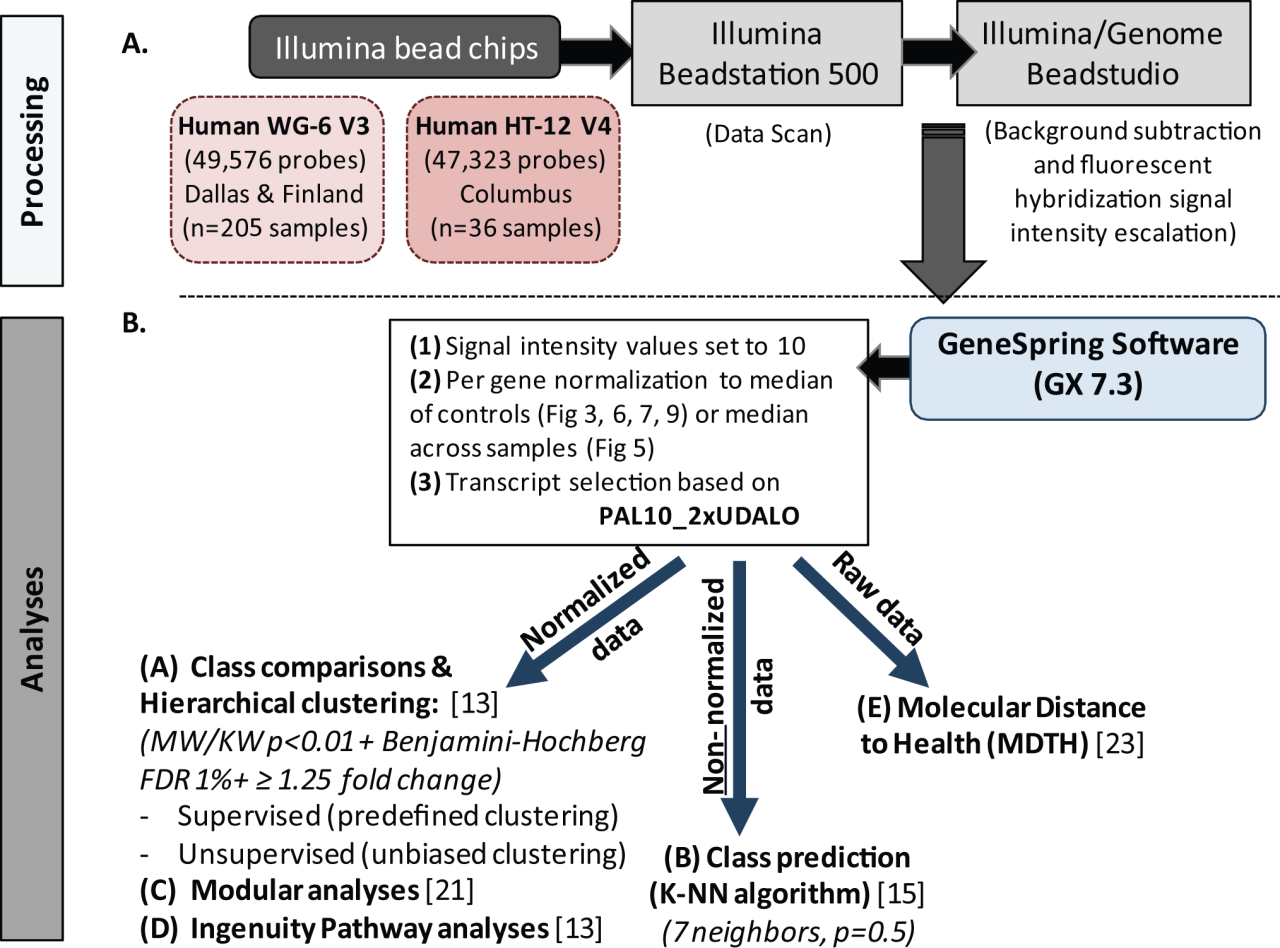
Microarray processing and statistical analyses. (A) Extracted and processed RNA was hybridized into Illumina human beadchips (205 samples into Human WG-6 v3 beadchips and 36 in to Human HT-12 v4) and scanned on the Illumina Beadstation 500, and fluorescent hybridization signals were assessed and scaled using Illumina BeadStudio. (B) For analysis we used GeneSpring software, IPA, modular analyses, and MDTH analysis. Depending on the type of analysis, either normalized, non-normalized, or raw data were used. FDR, false discovery rate; KW, Kruskal-Wallis test; MW, Mann-Whitney test.

Illumina BeadStudio/GenomeStudio software was used to subtract background and scale average samples' signal intensity, and GeneSpring GX 7.3 software was used to perform further normalization and analyses (Figure 2) [13],[15],[16].

#### Data import and normalization

First, all signal intensity values less than ten fluorescent units were set to equal ten. Next, per-gene normalization was applied by dividing the signal intensity of each probe in each sample by the median intensity for that probe across the median of each control group, except for the classifier analysis, where signals were normalized to the median intensity of that probe across all samples. These normalized data were used for all downstream analyses except for the identification of classifier genes, for which we used non-normalized data, and the assessment of molecular distance to health, for which we used raw data (Figure 2). Transcripts were first selected if they were present in ≥10% (PAL10%) of all samples, had a signal precision <0.01, and had a minimum of 2-fold expression up or down (2xUDALO) change compared with the median intensity across all samples [13]. Thus, the PAL10_2xUDALO, or quality control, gene list composed of 15,530 transcripts was used as the starting point for the downstream analysis (Figure S1: *n* = 241 samples; RSV, *n* = 156 [acute, *n* = 135; follow-up, *n* = 21], influenza, *n* = 16, HRV, *n* = 30, and healthy controls, *n* = 39). Then, we followed the strategy outlined below.

#### Data analysis

For supervised analysis (comparative analyses between predefined sample groups), we used GeneSpring software to perform Mann-Whitney or Kruskal-Wallis tests (*p*<0.01) for comparisons across study groups, followed by Benjamini-Hochberg multiple test correction (false discovery rate set at 1%) and a ≥1.25-fold change filter in expression level relative to the control group [13],[17]–[19]. The list of transcripts generated using the statistical filtering and class comparisons detailed above were then used for hierarchical clustering (of genes and patient samples). Unsupervised clustering (unbiased grouping of samples based on their molecular profile without prior knowledge of sample classification) was applied to the independent validation sets A and B [13]. For hierarchical clustering of genes, we used the Pearson correlation similarity measure with an average-linkage-clustering algorithm implemented in GeneSpring that organizes vertically all transcripts according to their trend of expression across all samples. The vertical expression profiles so generated were then hierarchically clustered by participant sample, on the basis of the similarity of their expression profiles and without prior knowledge of their classification, which now grouped participant samples into horizontally presented clusters on the basis of the similarity of their expression profiles. For this stage, we used the Spearman's rank correlation/distance similarity measure. By examining the cluster membership we could both assess whether the samples were grouping according to known factors (disease status, infecting virus, demographic features) and discover whether there were unknown subclasses within the dataset [14],[20].

Class prediction using the *k*-nearest neighbor (K-NN) algorithm, one of the tools available in GeneSpring, with seven neighbors and a *p*-value ratio cutoff of 0.5 was applied to identify the top-ranked genes that best discriminated among the three viral infections. For the class prediction analyses, we first generated a gene list composed of the transcripts differentially expressed (Kruskal-Wallis *p*<0.01 and Benjamini-Hochberg false discovery rate) among the three viral infections included in the training set. Using those transcripts a prediction model was refined by cross-validation on the training set. This model was then used to predict the classification of samples in the independent test set. Where no prediction was made, this was recorded as an indeterminate result. *p*-Values were determined using the two-sided Fisher's exact test [13],[15].

Functional analyses were performed using modular analysis. This is a systems scale strategy for microarray analysis that has identified transcriptional modules formed by genes coordinately expressed across multiple disease datasets, thus allowing functional interpretation of the microarray data into biologically useful information. A detailed account of this module-based mining analysis strategy has been reported elsewhere [13],[14],[21],[22].

Ingenuity Pathways Analysis (IPA) (Ingenuity Systems) was used to identify the top canonical pathways and networks. The significance of the association between the dataset and the canonical pathway was measured using Fisher's exact test. This program was also used to map the canonical network and overlay it with expression data from the dataset.

Molecular distance to health (MDTH), a metric that converts the global transcriptional perturbation of each sample into an objective score indicating the degree of transcriptional perturbation of a given sample compared with a healthy baseline, was calculated and correlated with parameters of disease severity. This analysis essentially consists of carrying out outlier analyses on a gene-by-gene basis, where the dispersion of the expression values found in the baseline samples (controls) is used to determine whether the expression value of a given sample (i.e., RSV patient) lies inside or outside two standard deviations of the mean value for the healthy controls, as described elsewhere [13],[14],[23].

Statistical analyses of other continuous and categorical variables were performed using Graph Pad Prism version 5. The Bonferroni correction was used to account for multiple statistical comparisons across all analyses. For sample size calculation, best practices in the microarray field dictate utilization of at least two independent sets of samples for the purpose of validating candidate signatures (or profiles). In previous studies in individuals with acute infections, others and we obtained robust profiles using groups with at least 20–30 participants each for the training and test sets [15],[20],[21],[24].

## Results

### Blood RSV Transcriptional Profile Is Characterized by Overexpression of Neutrophil and Myeloid Genes and Suppression of B and T Cell Genes

**Figure 3:**
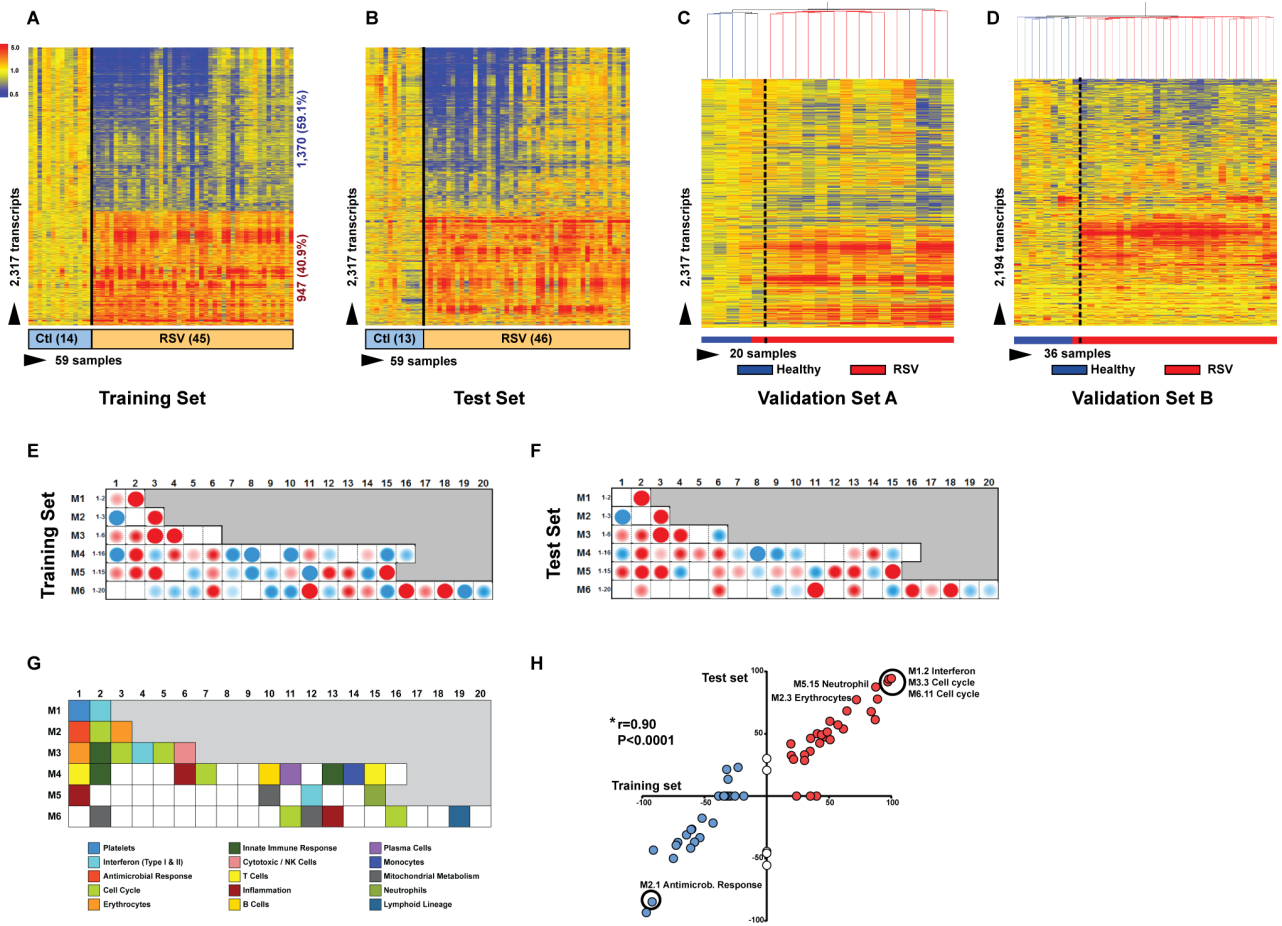
RSV transcriptional signature is characterized by overexpression of innate immunity and suppression of adaptive immunity. (A) Statistical group comparisons between children <2 y of age with RSV LRTI and healthy matched controls (Ctl) (Mann-Whitney test *p*<0.01, Benjamini-Hochberg multiple test correction and 1.25-fold change) yielded 2,317 significantly differentially expressed transcripts (training set; Dallas, Texas). Transcripts were organized by hierarchical clustering, where each row represents a single transcript and each column an individual participant. Normalized expression levels are indicated as overexpressed (red) or underexpressed (blue) compared to the median expression of healthy controls (yellow). (B) The same 2,317-transcript list applied to an independent set (test set; Dallas, Texas) of 46 children with RSV LRTI and 13 matched controls. (C) A third cohort of children with RSV LRTI was used as validation set A (Turku, Finland). Unsupervised hierarchical clustering of the 2,317 transcripts grouped all RSV patients together (red bar) except for the only patient who was diagnosed in the outpatient setting, who clustered with the controls. Dotted line indicates the cluster separation. (D) A fourth cohort of 28 infants with RSV and eight matched controls was used as validation set B (Columbus, Ohio) and was analyzed in a different gene chip (Illumina Human HT-12 v4). Unsupervised clustering of the 2,194 transcripts (123 transcripts were not present in this new gene chip) segregated patients and controls with high accuracy. Dotted line indicates the cluster separation. (E and F) Average modular transcriptional fingerprint for RSV LRTI in the training (E) and test (F) sets. Colored spots represent the percentage of significantly overexpressed (red) or underexpressed (blue) transcripts within a module in patients with RSV infection compared to controls (see [G] for module map key). Blank modules indicate no significant differences between patients and controls. Patients with RSV LRTI demonstrated significant overexpression of modules related to erythrocytes (M2.3, M3.1), platelets (M1.1), and cell cycle (M3.3, M4.7, M6.11, M6.16), and to innate immunity including interferon (M1.2, M3.4, M5.12), monocytes (M4.14), neutrophils (M5.15), innate immune responses (M3.2, M4.2), and inflammation (M4.6, M5.1, M6.13). Conversely, genes related to adaptive immunity: T cells (M4.1, M4.15), B cells (M4.10), lymphoid lineage (M6.19), cytotoxicity/NK cells (M3.6), and antimicrobial response (M2.1) were significantly underexpressed. (G) Key to the functional interpretation of each transcriptional module (M): module sets 1 to 6 are indicated on the *y*-axis, and module numbers within each set are indicated on the *x*-axis. (H) Scatter plot correlating (Spearman's *r*) percentage of modular expression between the training (*x*-axis) and the test (*y*-axis) sets. The interferon module (M1.2) and the antimicrobial response module (M2.1) were the most highly correlated between the training and the test sets.

To define the systemic host immune response to RSV infection, patients were divided into training, test, and validation sets (Table 1). Statistical group comparisons identified 2,317 differentially regulated transcripts between 45 infants with RSV and 14 healthy matched controls in the first group of patients in Dallas, Texas (training set; Figure 3A). Fifty-nine percent of transcripts were underexpressed, and 41% overexpressed. While the top ten overexpressed genes included genes related to interferon (*IFI27*) and neutrophil function (*DEFA1* and *ELA2*), the top underexpressed gene was related to immunosuppression (*COAS2*) (Table S2). This signature was validated in a second independent group of 46 infants with RSV and 13 controls enrolled in Dallas, Texas (test set; Figure 3B). Patients' demographic and clinical characteristics were comparable in both sets (Tables 1 and 2). A third group of 16 infants with RSV and four matched controls (validation set A) were enrolled in Turku, Finland. Unsupervised hierarchical clustering of the 2,317 transcripts previously identified was applied to this cohort, and the analysis grouped 15 of 16 infants with RSV together. The infant who clustered with the healthy control group was the only child in this cohort of 107 RSV-infected infants that was diagnosed as an outpatient and did not require hospitalization (Figure 3C). Lastly, we enrolled a fourth cohort (validation set B) composed of 28 infants with RSV LRTI and eight matched controls in Columbus, Ohio. These patients were analyzed using a different microarray chip from that used in the other cohorts, and yet, unsupervised hierarchical clustering of the signature identified in the training set grouped all but one RSV patient together (Figure 3D).

**Table 1:** Demographic and laboratory parameters in children with RSV LRTI.

Parameter	Training Set (Dallas, Texas)			Test Set (Dallas, Texas)			Validation Set A (Turku, Finland)			Validation Set B (Columbus, Ohio)		
	Patients (*n* = 45)	Controls (*n* = 14)	*p*-Value	Patients (*n* = 46)	Controls (*n* = 13)	*p*-Value	Patients (*n* = 16)	Controls (*n* = 4)	*p*-Value	Patients (*n* = 28)	Controls (*n* = 8)	*p*-Value
**Age (months)**	2.33[1.57–6.32]	4.93[3.96–6.72]	0.08	1.65[0.69–2.93]	9.80[2.45–13.87]	0.001	6.47[4.01–12.72]	9.45[6.77–12.13]	0.43	2.91[1.62–4.31]	5.38[1.30–12.16]	0.48
**Gender**			0.13			0.76			1			0.22
Male	24 (53)	4 (29)		22 (48)	7 (54)		7 (44)	2 (50)		17 (61)	7 (87)	
Female	21 (47)	10 (71)		24 (52)	6 (46)		9 (56)	2 (50)		11 (39)	1 (13)	
**Race/ethnicity**			0.62			0.13	16 (100)	4 (100)	1			0.93
White	9 (20)	3 (22)		10 (22)	1 (8)					22 (79)	7 (87)	
Black	2 (4)	2 (14)		4 (9)	4 (31)					3 (11)	1 (13)	
Hispanic	31 (69)	8 (57)		29 (63)	8 (61)					2 (7)	—	
Other^a^	3 (7)	1 (7)		3 (6)	—					1 (3)	—	
**White blood cells/mm^3^**	9.95[7.72–11.68]	14.05[10.73–15.45]	<0.01	10.85[8.75–14.85]	10.30[8.30–10.70]	0.20	10.00[7.27–12.63]	8.90[6.60–10.40]	0.43	12.00[9.15–16.60]	7.90[7.62–9.02]	0.01
**Neutrophil percent**	18.90[11.25–24.00]	22.15[16.88–25.83]	0.15	22.00[13.75–31.75]	28.00[19.20–29.95]	0.50	N/A	N/A	—	29.00[17.50–31.50]	22.50[14.00–25.75]	0.17
**Lymphocyte percent**	67.50[58.0–75.00]	70.00[66.20–75.80]	0.18	60.00[53.00–70.75]	63.30[60.50–71.80]	0.14	N/A	N/A	—	53.00[43.00–61.00]	70.50[58.50–75.75]	0.01
**Monocyte percent**	9.50[6.00–13.00]	5.00[3.25–6.97]	<0.01	8.50[6.00–12.00]	5.20[4.50–7.50]	0.01	N/A	N/A	—	10.00[8.00–14.00]	7.50[4.25–11.25]	0.11

Data reported as median [IQR] or number (percent). *p*-Values from Mann-Whitney test or Fisher's exact test and Chi square for continuous and categorical variables, respectively.

a “Other” includes patients of Asian origin or mixed race.

N/A = not available

**Table 2:** Clinical parameters and radiologic findings in children with RSV LRTI.

Parameter	Training Set (*n* = 45)	Test Set (*n* = 46)	Validation Set A (*n* = 16)	Validation Set B (*n* = 28)	*p*-Value 1	*p*-Value 2	*p*-Value 3
**Symptoms before admission (days)**					0.09	<0.01	<0.01
Median [IQR]	3.0 [2.0–3.0]	3.0 [2.0–5.0]	5.0 [3.0–7.0]	4.0 [3.0–5.0]			
Median [range]	3.0 [1.0–14.0]	3.0 [1.0–10.0]	5.0 [2.0–14.0]	4.0 [2.0–17.0]			
**Symptoms before sample collection (days)**					0.42	0.34	0.66
Median [IQR]	5.0 [4.0–6.5]	6.0 [4.0–7.25]	5.0 [3.0–7.0]	5.0 [4.0–6.0]			
Median [range]	5.0 [2.0–17.0]	6.0 [2.0–11.0]	5.0 [2.0–14.0]	5.0 [3.0–20.0]			
**Supplemental O_2_**							
Number (percent) requiring	29 (65)	31 (67)	3 (19)	25 (89)	0.82	<0.01	0.02
Median [IQR] duration (days)	4.0 [2.0–8.0]	3.0 [2.0–5.0]	1.0 [0.5–3.0]	3.0 [2.0–4.0]	0.51	0.08	0.06
**Intensive care unit**						N/A	
Number (percent) requiring	10 (22)	12 (26)	0 (0)	9 (32)	0.80		0.41
Median [IQR] duration (days)	7.5 [5.25–9.25]	5.0 [1.50–9.50]	N/A	3.0 [1.0–4.5]	0.30		<0.01
**Invasive ventilatory support/CPAP**						N/A	
Number (percent) requiring	9 (20)	11 (24)	0 (0)	8 (29)	0.80		0.41
Median [IQR] duration (days)	6.5 [5.25–7.75]	4.0 [2.0–9.0]	N/A	3.0 [1.1–4.0]	0.58		<0.01
**Median [IQR] length of stay (days)**	4.0 [2.5–8.0]	4.0 [2.75–7.0]	2.0 [1.0–2.25]	2.0 [2.0–4.0]	0.72	<0.001	<0.01
**Median [IQR] CDSS**	4.0 [1.0–8.5]	5.0 [2.0–9.5]	4.5 [2.0–8.75]	6.0 [4.0–8.0]	0.62	0.54	0.20
**Number (percent) chest X-ray performed**	36 (80)	39 (85)	N/A	24 (86)	0.59	N/A	0.75
**Number (percent) chest X-ray reading**			N/A		0.44	N/A	0.52
No pathologic findings	5 (14)	2 (5)		1 (4)			
Bronchial wall thickening	18 (50)	17 (44)		12 (50)			
Atelectasis	9 (25)	14 (36)		9 (38)			
Lobar consolidation	4 (11)	6 (15)		2 (8)			

*p*-Values from Mann-Whitney test or Fisher's exact test and Chi square for continuous and categorical variables, respectively: *p*-value 1 (training versus test); *p*-value 2 (training versus validation set A); *p*-value 3 (training versus validation set B).

CPAP, continuous positive airway pressure; N/A, not applicable.

**Figure 4:**
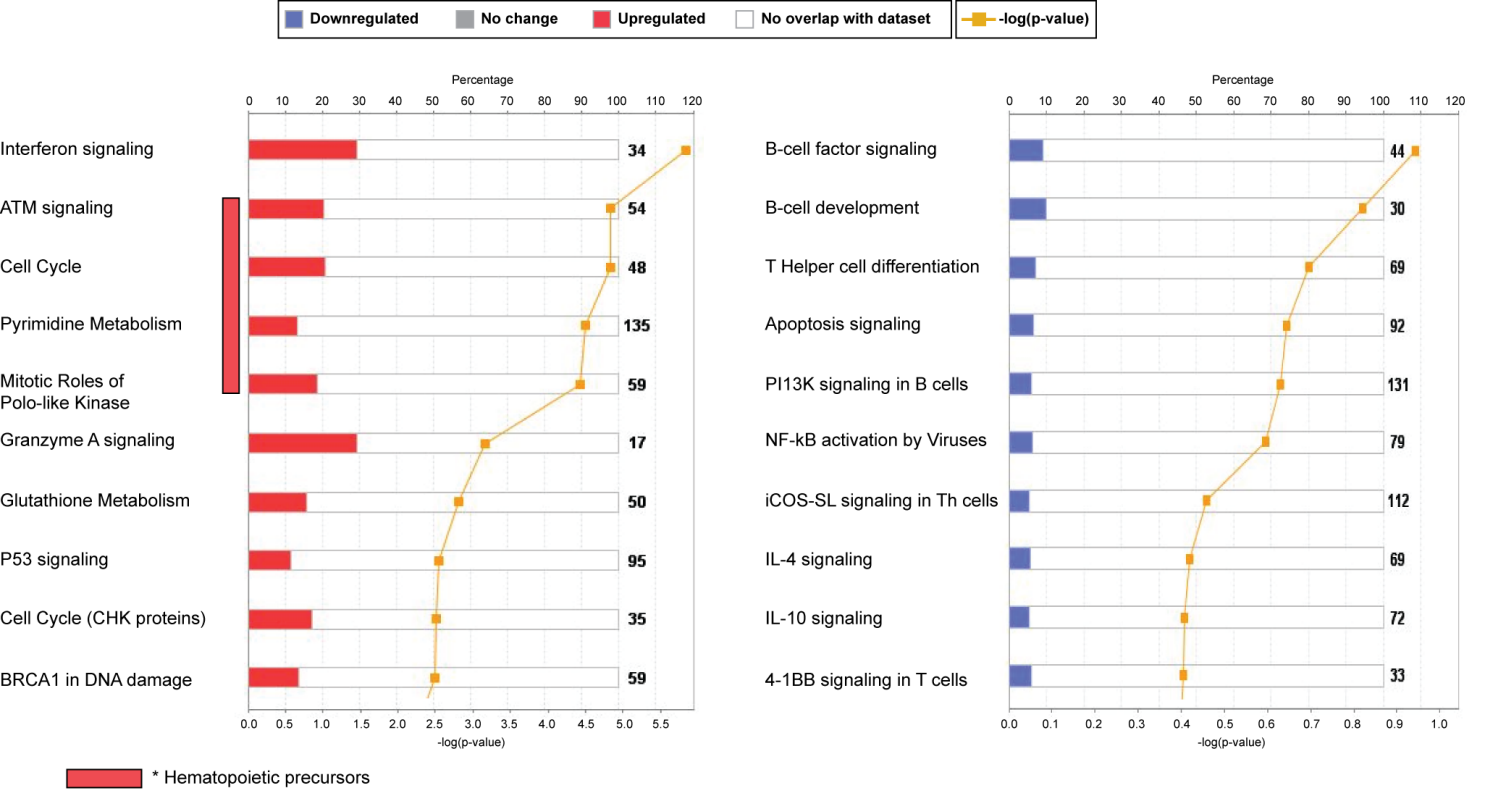
Top canonical pathways expressed in children with RSV LRTI. IPA showed that the interferon-signaling pathway followed by genes related to cell cycle and hematopoietic precursors (ATM) were the most upregulated pathways, while the B cell and T cell signaling pathways were the most downregulated pathways, confirming our previous results using modular-level analyses. iCOS-SL in Th cells, inducible costimulator signaling in T helper cells.

To characterize the biological significance of the blood RSV signature, we applied an analytical framework of 62 transcriptional modules (Figure 3G) [14],[21],[22]. Module maps were derived independently for the training (Figure 3E) and test (Figure 3F) sets, using their respective healthy control groups as reference. Patients with RSV LRTI demonstrated significant overexpression of modules related to inflammation, erythrocytes, platelets, cell cycle, and innate immunity including interferon, monocyte, neutrophil, and innate immune response genes. Conversely, genes regulating adaptive immunity involving T cells, B cells, lymphoid lineage, cytotoxic/natural killer (NK) cells, and antimicrobial response were significantly underexpressed. These findings were validated in the test set, as demonstrated by a significant correlation of module expression between both sets (*r* = 0.90, *p*<0.0001; Figure 3H). The interferon module (overexpressed) and the antimicrobial response module (underexpressed) were the most highly correlated modules. IPA confirmed the over- and underexpression of the innate and adaptive immune responses, respectively (Figure 4).

### RSV Induces a Unique Transcriptional Profile in Peripheral Blood

**Figure 5:**
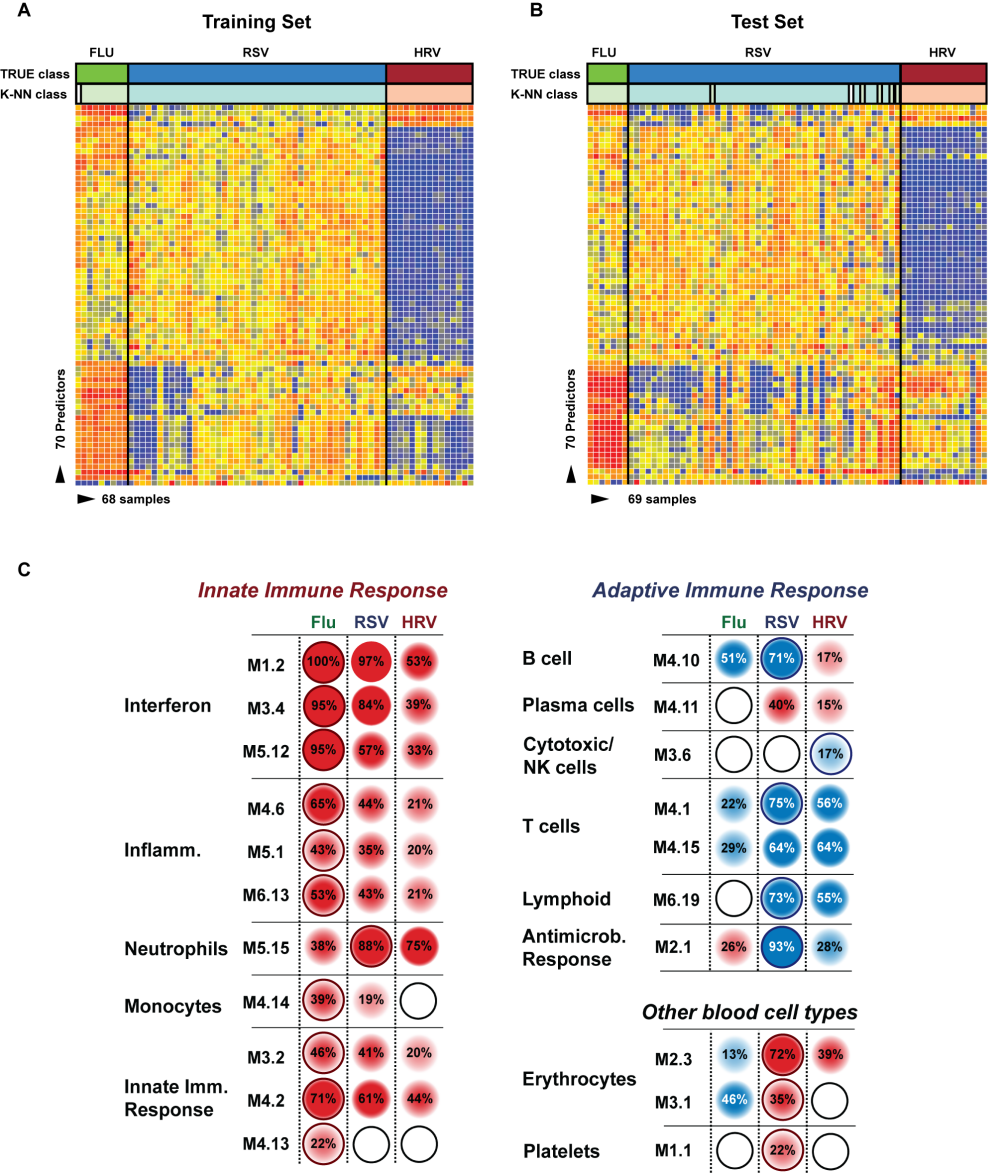
Transcriptional profiles from children with influenza, RSV, and HRV LRTI. (A) A supervised learning K-NN algorithm with seven neighbors and a *p*-value ratio cutoff of 0.5 was used to identify the 70 top-ranked genes that best discriminated RSV from HRV and influenza LRTI. Using the 70 classifier genes, leave-one-out cross-validation of the training set correctly classified 67 of the 68 samples (influenza [*n* = 9; green]; RSV [*n* = 44, blue]; HRV [*n* = 15, burgundy]) (98% accuracy). The patient sample that was not classified correctly belonged to an infant <6 mo old with mild influenza A LRTI (#207). Predicted class is indicated by light-colored rectangles. (B) The 70 classifier genes were cross-validated on an independent set of 69 new patients (test set; influenza *n* = 7; RSV *n* = 47; HRV *n* = 15). The algorithm correctly categorized 63 of the 69 new patient samples (91% accuracy). Five samples (#22, #38, #45, #46, #49) from infants with RSV were misclassified as influenza. These five RSV patients demonstrated overexpression of the 18 top overexpressed influenza classifier genes, which was not demonstrated in the rest of the RSV cohort (Table 2). One patient with mild RSV LRTI (#47) was not predicted. (C) Mean modular transcriptional fingerprint for influenza (*n* = 16 and 10 matched controls), RSV (*n* = 44 and 14 matched controls), and HRV LRTI (*n* = 30 and 14 matched controls). Overall, children with HRV infection demonstrated a milder activation of the innate and adaptive immune responses, compared with children with influenza or RSV infection. Children with influenza displayed a stronger activation of genes related to interferon (M1.2, M3.4, M5.12), inflammation (M4.6, M5.1, M6.13), monocytes (M4.14), and innate immune response (M3.2, M4.2, M4.13) compared with children with RSV or HRV. Several type I interferon (*IFIH1, IFIT1–5, STAT2, MX1*) and type II interferon (*IFI16, CXCL10, CCL8, GBP1–5, STAT1, SOCS1*) genes were expressed only in influenza and RSV infection (Table S3). In addition, the magnitude of the type I interferon (*IFI44, IFI44L, OAS2, IRF7*) and type II interferon (*IFI35, IFITM1–3*) response present was 2- to 22-fold higher in children with influenza compared with children with RSV or HRV. Similarly, genes related to inflammation, monocytes, and innate immune response were greatly overexpressed in children with influenza compared to children with RSV or HRV LRTI. Neutrophil-related genes (M5.15) such as *CEACAM6*, *DEFA4*, *MPO*, and *MMP8* were significantly overexpressed in RSV infection, followed by HRV infection and, at a lower level, influenza infection. *DEFA1*, *DEFA3*, *ELA2*, *CEACAM8*, and *AZU1* were expressed only in RSV and HRV infection. Three genes were solely and significantly expressed in RSV infection but not in influenza or HRV infection: *LTF*, *RETN*, which binds to *DEFA1*, and the scavenger receptor *OLR1*. On the other hand, the suppression of genes related to B cells (M4.10), T cells (M4.1, M4.15), lymphoid lineage (M6.19), and antimicrobial response (M2.1) observed in RSV infection was significantly milder or not present in children with influenza or HRV LRTI. The outer dark circles highlight the disease group (influenza, RSV, or HRV) with greater (red) or lower (blue) modular activation.

To determine whether the systemic host immune response to RSV infection was specific we applied a K-NN class prediction algorithm. This algorithm yielded 70 classifier genes that best discriminated RSV from HRV and influenza LRTI in two independent cohorts of patients (Tables 3 and 4). Using the 70 classifier genes, leave-one-out cross-validation of the training set correctly classified 67 of 68 samples (98% accuracy; Figure 5A). In the validation analysis (test set), classifier genes correctly categorized 63 of 69 new patient samples (91% accuracy; Figure 5B). Thus, the K-NN algorithm demonstrated a sensitivity of 94% (95% CI 87%–98%) and a specificity of 98% (95% CI 88%–99%) (Table 5).

**Table 3:** List of classifier genes that best discriminate RSV from influenza A and rhinovirus LRTI.

Category	Gene	Influenza	HRV	RSV
**Viral induced**	*RGD1309995*	1.25	0.25	1.10
	*COG3*	1.16	0.50	1.09
	*GTF2H1*	1.29	0.35	1.09
	*HECA*	1.00	0.40	1.07
	*RAB6A*	0.87	0.24	1.12
	*PTGES3*	0.92	0.52	1.12
	*RPL10A*	0.79	0.69	1.18
	TRIM14*	3.17	1.04	0.83
	*YY1*	0.90	0.39	1.11
**Immune related**	*BIRC3*	2.00	1.53	0.77
	*HMGB1*	1.30	0.40	1.10
	*HMGB1*	0.96	0.50	1.10
	*FLI1*	1.15	0.25	1.07
	*TPP2*	1.33	0.53	1.04
	*IGBP1P1*	1.04	0.21	1.18
	*CAPZA1*	0.87	0.35	1.20
	*TANK*	1.06	0.16	1.12
	TNFSF14*	2.15	0.77	1.03
	SYAP1*	2.57	0.73	1.02
	*IKBKB*	1.34	0.32	1.17
	*RANBP9*	0.89	0.30	1.12
**Apoptosis**	*RHOT1*	2.10	0.71	1.09
	*PAK2*	2.06	1.72	0.87
	GSDMB*	2.98	1.18	0.79
	WWOX*	2.56	0.81	1.00
	ATRX*	2.04	1.13	0.85
	*PPP2CB*	1.18	0.31	1.14
	*TAF9*	1.07	0.30	1.16
**Cell function: protein binding/synthesis**	ERGIC1*	3.22	0.79	0.99
	*FRA10AC1*	0.80	0.66	1.14
	*GSKIP*	0.78	0.52	1.10
	*FKSG17*	0.83	0.37	1.18
	CSNK1G2*	1.75	0.54	1.02
	*OSTC*	0.85	0.39	1.09
	PICALM*	2.04	0.47	1.11
	*CCZ1*	0.97	0.54	1.16
**Cell function: cell growth/cycle**	*KIAA1751*	2.07	0.79	0.90
	*TTDN1*	0.96	0.29	1.17
	*MINA*	1.29	0.52	1.09
	SPATA13*	1.87	0.83	1.00
**Cell function: zinc metabolism**	*OMA1*	1.38	0.28	1.08
	*TRMT1L*	1.27	0.19	1.11
	*POGZ*	2.66	1.40	0.79
	RNF213*	2.95	0.66	1.06
	*MBNL3*	0.68	1.50	1.01
	ZNF557*	2.39	0.74	1.00
**Cell function: other functions**	CCBE1*	2.58	0.81	0.96
	*NUS1*	1.06	0.42	1.23
	LMBRD1*	1.63	1.24	0.81
	*SEPT7*	1.09	0.23	1.09
	*HEXDC*	1.88	0.48	1.10
	PLIN5*	2.45	0.82	0.98
	SLC25A20*	3.19	0.97	1.00
	*NIPBL*	1.59	0.61	1.04
	SNRNP35*	1.42	0.44	1.05
	*RPS28*	0.99	0.40	1.13
	*COPS2*	0.95	0.38	1.18
	*TUBD1*	1.21	0.42	1.11
	*NR2C2*	2.54	1.41	0.69
	*GSG1L*	1.15	2.25	0.83
	NBPF1*	3.17	1.49	0.73
	*ANAPC1*	1.69	1.26	0.84

Numeric values represent the median expression values per transcript per study group in the training and cross-validation sets. Genes in bold and followed by an asterisk are the top 18 transcripts significantly overexpressed in influenza A infection (Figure 5A and 5B) compared with RSV and HRV.

**Table 4:** Demographic characteristics of children with RSV, rhinovirus, and influenza LRTI.

Characteristic	Training Set (*n* = 68)				Test Set (*n* = 69)			
	RSV (*n* = 44)	HRV (*n* = 15)	Influenza (*n* = 9)	*p*-Value	RSV (*n* = 47)	HRV (*n* = 15)	Influenza (*n* = 7)	*p*-Value
**Age (months)**	1.90[1.14–2.93]	2.77[1.63–9.87]	6.33[2.03–7.85]	0.08^a^	2.13[1.27–4.43]	1.70[0.70–5.07]	7.1[2.33–14.87]	0.04^a^
**Sex (male: female)**	23M: 21 F	10M: 5 F	5M: 4 F	0.62^b^	23M: 24 F	9M: 6 F	2M: 5 F	0.38^b^
**CDSS**	6.0 [2.0–12.75]	6.0 [4.0–8.0]	3.0 [1.0–8.5]	0.24^a^	4.0 [2.0–7.0]	4.0 [2.0–8.0]	2.5 [1.0–12.0]	0.77^a^
**Length of hospitalization (days)**	5.0 [3.0–7.0]	4.0 [3.0–9.0]	4.0 [2.5–7.0]	0.93^a^	3.0 [2.0–7.0]	3.0 [3.0–5.0]	2.0 [0.0–2.0]	0.005^a^

Data reported as median [IQR] except for sex.

a Kruskal-Wallis one-way analysis of variance.

b Chi square test.

**Table 5:** Results from the K-NN class prediction algorithm.

Classification	Training Set				Test Set			
	RSV(*n* = 44)	HRV(*n* = 15)	Influenza(*n* = 9)	Total(*n* = 68)	RSV(*n* = 47)	HRV(*n* = 15)	Influenza(*n* = 7)	Total(*n* = 69)
Correct number of patients (percent)	44	15	8	67 (98.5%)	41	15	7	63 (91.3%)
Incorrect number of patients (percent)	0	0	1	1 (1.5%)	5	0	0	5 (7.2%)
Not classified (percent)	0	0	0	0 (0%)	1	0	0	1 (1.5%)
	**Training Set**				**Test Set**			
Sensitivity (95% CI)	100% (92%–100%)				89% (76%–96%)			
Specificity (95% CI)	96% (79%–99%)				100% (85%–100%)			
	**Combined**							
Sensitivity combined^a^ (95% CI)	94% (87%–98%)							
Specificity combined^a^ (95% CI)	98% (88%–99%)							

a Training and test sets.

N/A, not applicable.

To further define the differences in immune profiles among these viruses, we compared the modular fingerprint derived from patients with RSV, influenza, and HRV and from a cohort of healthy matched controls for each viral infection. Although influenza, RSV, and HRV shared common pathways, both the degree of activation/suppression and the expression of specific immune-related genes were markedly different among them. HRV induced milder activation of innate and adaptive immune responses while influenza stimulated a stronger activation of interferon (qualitatively and quantitatively; Table S3), inflammation, monocyte, and innate immune response genes compared with RSV and HRV. Neutrophil-related genes were significantly overexpressed in patients with RSV, followed by patients with HRV, and were at a lower level in patients with influenza LRTI. Lastly, RSV was associated with marked suppression of B cell, T cell, lymphoid lineage, and antimicrobial response genes; this suppression was significantly milder or absent in children with influenza and HRV LRTI (Figure 5C).

### Blood Host Immune Profiles Remain Altered 1 mo after Acute RSV LRTI

**Figure 6:**
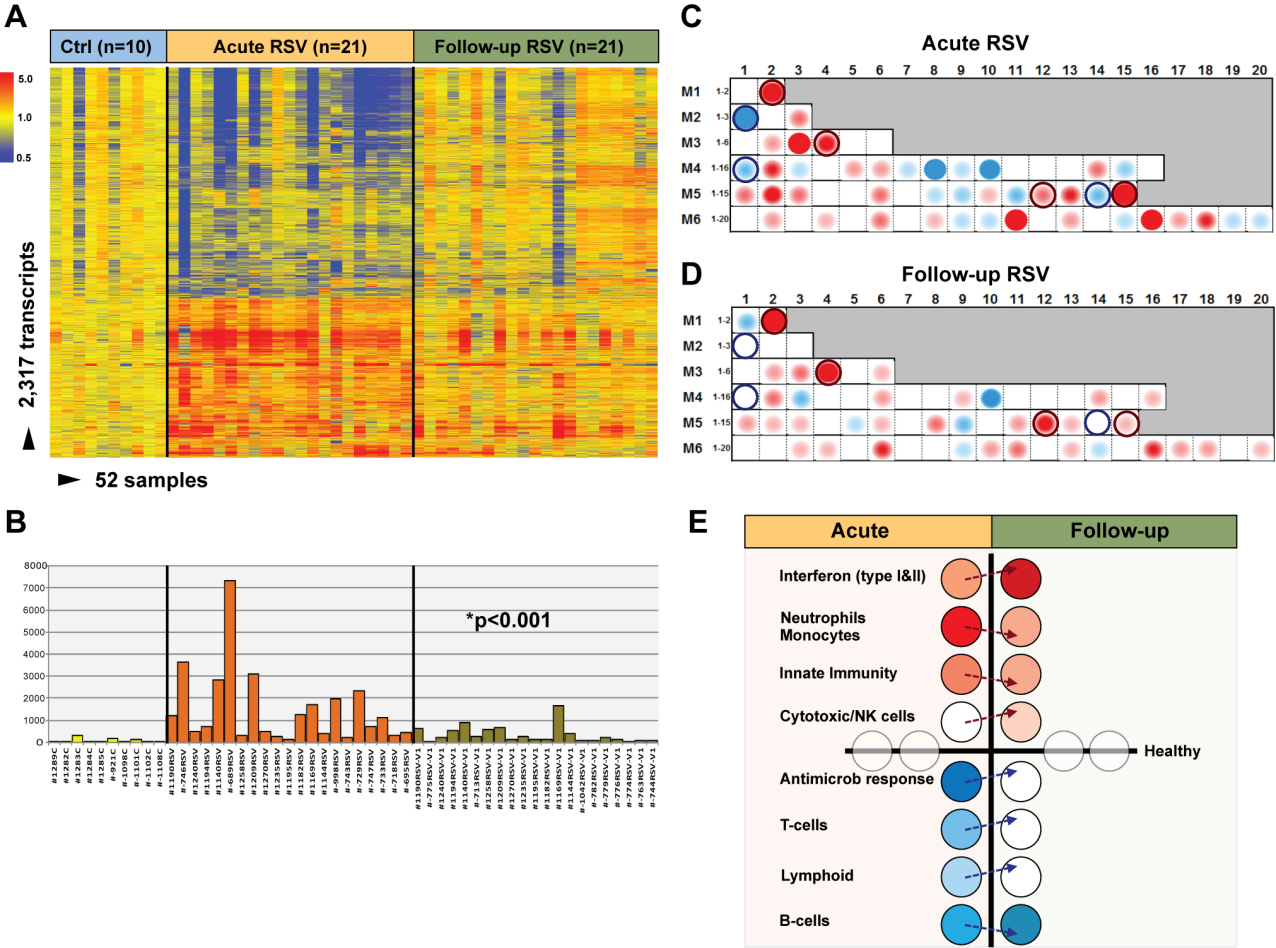
Blood host immune profiles remain altered 1 mo after acute RSV LRTI. (A) Samples from 21 infants with RSV LRTI were obtained 1 mo after the acute hospitalization. Hierarchical clustering of control (Ctrl), acute, and follow-up samples reflected the heterogeneity observed during the acute disease. (B) MDTH scores per patient are represented by bars (yellow: healthy controls; orange: acute RSV LRTI; green: RSV follow-up) underneath the expression profile for that specific sample in (A). Wilcoxon rank paired *t*-test demonstrated significantly lower MDTH scores at follow-up compared with during the acute disease. (C and D) Average modular transcriptional fingerprint for acute RSV LRTI (C) and follow-up (D). Colored spots represent the percentage of significantly overexpressed (red) or underexpressed (blue) transcripts within a module in patients with RSV infection compared to controls (see Figure 3G for module map key). Circle rings highlight modules with greater changes from the acute to the follow-up visit. (E) Analysis of modular activation during acute RSV LRTI and follow-up revealed overexpression of interferon-related genes (M1.2, M3.4, M5.12) at a greater level at follow-up than during acute disease (54% in acute RSV versus 78% at follow-up; *p*<0.001). This effect was specifically observed in type-I interferon (*TRIM25*) but mostly in interferon-γ-related genes (*BTN3A1, TAP2, SP100, SP110, NUB1*). Genes related to neutrophils (M5.15), monocytes (M4.14), and innate immunity (M3.2, M4.2) that were overexpressed during acute disease showed decreased expression at follow-up. Cytotoxic/NK cell (M3.6) genes were significantly overexpressed at follow-up compared with the acute disease. B cell (M4.10) genes remained underexpressed over time, but genes related to T cells (M4.1, M4.15), lymphoid lineage (M6.19), and antimicrobial response (M2.1), which were underexpressed during acute RSV, reached expression levels comparable to those observed in healthy controls (grey circles) at follow-up.

To determine whether and how the blood RSV signature evolved over time, samples from 21 infants (median age [IQR] 2.4 [0.9–7.1] mo) with mild (*n* = 14), moderate (*n* = 5), or severe disease (*n* = 2) were obtained median (IQR) 1.1 (0.9–2.1) mo after hospitalization. Analysis of these samples reflected the heterogeneity observed in those same patients during the acute disease (Figure 6A). MDTH scores [13],[23] were significantly decreased at follow-up visits compared with those elicited during the acute disease (*p*<0.001; Figure 6B). Although most of the patients followed had a relatively mild course during the acute hospitalization, modular analysis at follow-up revealed significant overexpression of interferon genes, at an even greater level than that observed during hospitalization (*p*<0.001). The overexpression of neutrophil, monocyte, and innate immunity genes observed during the acute disease faded over time. On the other hand, T cell, lymphoid lineage, and antimicrobial response genes, which were suppressed during the acute phase, reached expression levels comparable to those of controls at follow-up. B cell genes were persistently underexpressed during the acute and follow-up visits (Figure 6C–6E).

### Age Influences the Systemic Host Immune Response to RSV

**Figure 7:**
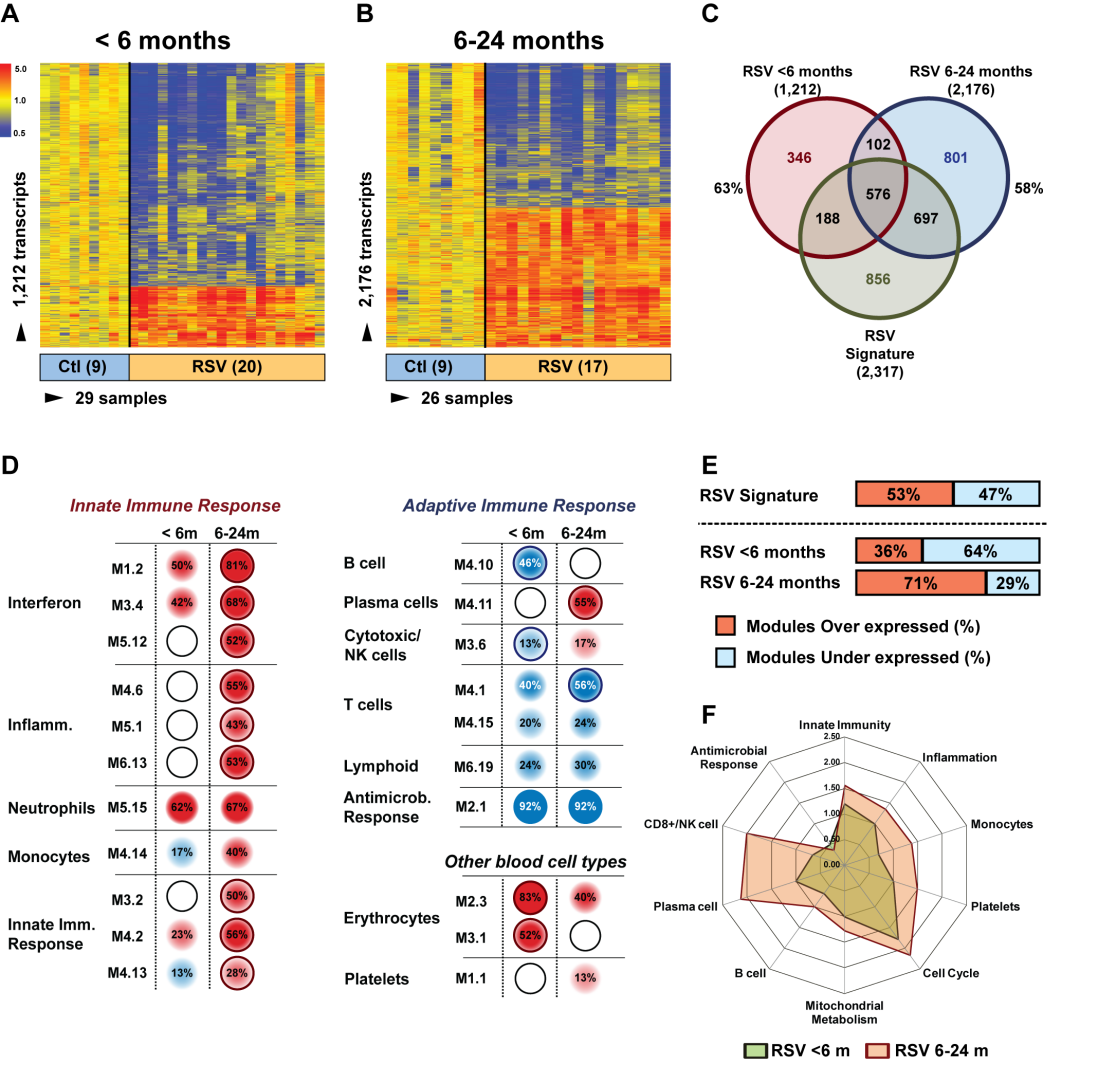
Age at the time of infection influences the host immune response to RSV. (A) Statistical group comparisons between 20 patients <6 mo of age and nine healthy matched controls (Ctl) yielded 1,212 significantly differentially expressed transcripts between the two groups. Of those, 952 (79%) transcripts were underexpressed. (B) The same type of analysis using 17 children with RSV LRTI at age 6–24 mo and nine healthy matched controls yielded 2,176 significantly differentially expressed transcripts, with 1,075 (49%) transcripts underexpressed. (C) Venn diagram displaying the overlap between the global RSV signature described in Figure 3A and the age-specific RSV gene expression profiles. (D) Modular analysis in the two age groups revealed a similar trend in the overexpression of neutrophil-related genes (M5.15) and the suppression of genes related to T cells (M4.1, M4.15), lymphoid lineage (M6.19), and antimicrobial response (M2.1). On the other hand, the overall activation of the innate immunity, interferon, and inflammatory response was decreased in infants <6 mo, and the adaptive immune response (B cells [M4.10], plasma cells [M4.11], and cytotoxic/NK cells [M3.6]) was further suppressed compared with children 6–24 mo of age. Circle rings indicate the modules within each group with greater over- or underexpression. (E) Horizontal bars illustrating the proportion of over- and underexpressed modules in infants <6 mo and children 6–24 mo of age in relation to the global RSV signature. (F) These differences are further illustrated in a spider graph representing the per-module median expression values of the significantly differentially expressed modules between the two age groups.

Next, we examined whether age influenced the systemic host response to RSV. To exclude the possibility that the differences observed were driven by demographic or clinical factors, we selected 20 infants <6 mo old with RSV LRTI who were matched for race, sex, and disease severity with 17 children 6–24 mo old with RSV LRTI and with nine healthy controls per age group (Table 6). We identified 1,212 significantly differentially expressed genes in children <6 mo (63% present in the RSV signature), and 2,176 significantly differentially expressed genes in children 6–24 mo (58% present in the RSV signature) compared with controls (Figure 7A–7C). Overall, the proportion of underexpressed genes was significantly greater in younger infants compared with the 6–24-mo age group (78% versus 49%; *p*<0.001). This greater suppression was demonstrated qualitatively and quantitatively at the modular level (Figure 7D and 7E). The overall activation of innate immunity and inflammation genes was less pronounced in infants <6 mo, and the adaptive immune response was further suppressed compared with older children. Differences in median expression values of the significant modules between age groups are shown in Figure 7F. Data regarding age differences in children with HRV and influenza LRTI are shown in Figure S2.

**Table 6:** Demographic characteristics and clinical disease severity in children with RSV LRTI.

Characteristic	Age 1–6 mo			Age 6–24 mo			*p*-Value^a^
	Patients^a^ (*n* = 20)	Controls (*n* = 9)	*p*-Value	Patients^a^ (*n* = 17)	Controls (*n* = 9)	*p*-Value	
**Age (months)**	2.4 [1.5–4.0]	3.9 [2.4–4.1]	0.22^b^	10.8 [8.0–17.3]	10.5 [8.9–14.8]	1.000^b^	<0.001
**Gender (male: female)**	11M: 9 F	4M: 5 F	1.0^c^	7M: 10 F	5M: 4 F	0.682^c^	0.51
**Race/ethnicity (number)**			0.61^d^			0.251^d^	0.79^d^
White	4	2		2	2		
Black	2	0		2	3		
Hispanic	14	7		13	4		
**CDSS**	2.5 [2.0–10.25]	N/A	—	2 [1.0–5.5]	N/A	—	0.59^b^
**Number (percent) requiring oxygen**	10 (50%)	N/A	—	10 (59%)	N/A	—	0.74^c^
**Length of hospital stay**	3.0 [2.0–8.0]	N/A	—	3.0 [2.0–4.5]	N/A	—	0.73^b^

Data reported as median [25%–75% interquartile range] unless otherwise indicated.

a Comparisons between children younger and older than 6 mo of age.

b Mann-Whitney test.

c Fisher's exact test.

d Chi square test.

N/A, not applicable.

### RSV Disease Severity Is Associated with Suppression of the Systemic Host Immune Response

To identify specific components of the host immune response that best correlated with the differences observed in RSV disease severity, we performed a comparative analysis according to clinical presentation. Children with RSV LRTI, matched for age, sex, and race, were classified as having mild (*n* = 20), moderate (*n* = 17), or severe disease (*n* = 16) using a CDSS (Table 7) [10].

**Table 7:** Demographic characteristics of children with mild, moderate, or severe RSV LRTI.

Parameter	Mild RSV (*n* = 20)	Moderate RSV (*n* = 17)	Severe RSV (*n* = 16)	Healthy (*n* = 10)	*p*-Value
**Age (months)**	2.28 [0.76–4.29]	2.27 [0.68–6.81]	1.90 [1.35–2.76]	4.03 [2.57–6.27]	0.13^a^
**Gender (male: female)**	10M: 10 F	7M: 10 F	9M: 7 F	4M: 6 F	0.79^b^
**Race/ethnicity (number)**					0.51^b^
White	4	7	5	4	
Black	0	0	0	0	
Hispanic	16	10	11	6	
**Length of hospital stay (days)**	2.0 [2.0–3.0]	5.0 [4.0–7.0]	11.0 [7.5–14.0]	N/A	<0.001^a^
**CDSS**	1.0 [1.0–2.0]	7.0 [6.5–8.0]	14.0 [13.0–14.0]	N/A	<0.001^a^

Data reported as median [IQR] unless otherwise indicated. CDSS: mild RSV, 0–5; moderate RSV, 6–10; severe RSV, 11–15.

a Kruskal-Wallis one-way analysis of variance.

b Chi square test.

N/A, not applicable.

**Figure 8:**
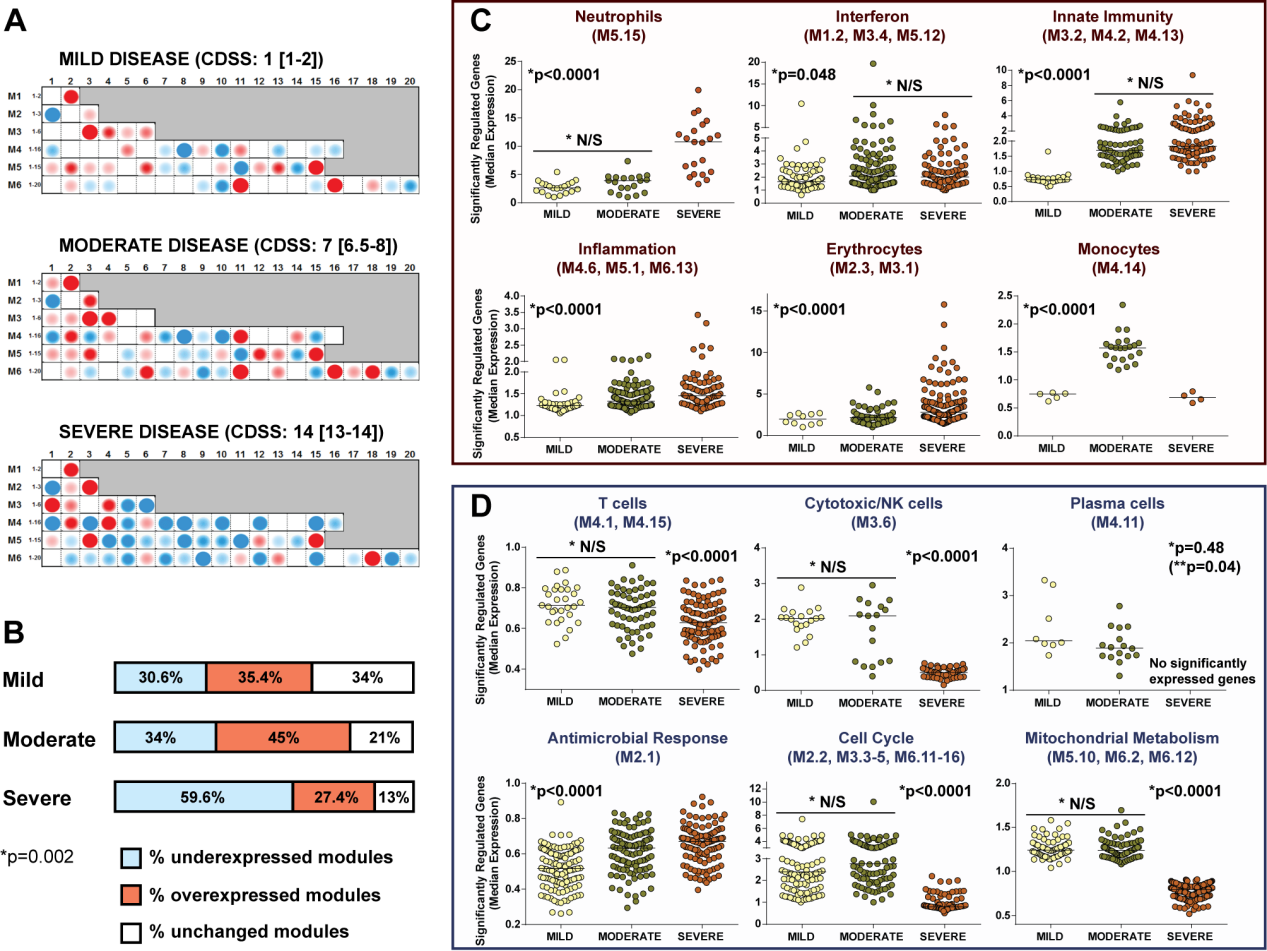
RSV disease severity is driven by greater suppression of the host immune response. (A) Modular fingerprints were independently derived from children with mild (*n* = 20), moderate (*n* = 17), or severe (*n* = 16) disease based on a CDSS, and from ten healthy controls. All RSV patients and controls were age and gender matched. Median (IQR) CDSS indicated for each disease severity group. (B) Horizontal bars illustrating the percentage of overexpressed, underexpressed, and unchanged modules compared to healthy controls for children with mild, moderate, and severe RSV LRTI (Chi square test *p* = 0.0025). (C and D) Significantly overexpressed (C) and underexpressed (D) modules in children with different degrees of clinical severity. Dots represent the median expression value for each individual transcript in all three disease severity groups (mild, moderate, and severe) per module or module aggregate sharing the same function. Genes related to interferon (M1.2, M3.4, M5.12) and innate immunity (M3.2, M4.2, M4.13) were significantly overexpressed in children with either moderate or severe RSV LRTI compared with children with mild disease. Overexpression of genes related to neutrophils (M5.15), inflammation (M4.6, M5.1, M6.13), and erythrocytes (M2.3, M3.1) significantly increased with disease severity. Children with severe RSV LRTI had significantly greater underexpression of genes related to T cells (M4.1, M4.15), cytotoxic/NK cells (M3.6), plasma cells (M4.11), cell cycle (M2.2, M3.3–5, M6.11–16), and mitochondrial metabolism (M5.10, M6.2, M6.12). Except for plasma cells, where adjusted (single asterisk) and unadjusted (double asterisk, in parentheses) *p*-values are displayed, all other *p*-values represent adjusted *p*-values (single asterisk) after applying the Bonferroni correction for multiple comparisons. N/S, not significant.

Modular analysis of children with severe RSV disease showed a significantly greater proportion of underexpressed modules than for children with moderate or mild disease (*p*<0.01; Figure 8A and 8B). The overexpression of neutrophil, inflammation, and erythrocyte genes increased significantly with disease severity. On the other hand, overexpression of interferon and innate immunity genes was similar in children with moderate and severe RSV LRTI, but greater than in children with mild disease (Figure 8C). B cell genes were consistently underexpressed in patients with mild, moderate, and severe RSV LRTI, while children with severe RSV LRTI demonstrated significantly greater underexpression of T cell, cytotoxic/NK cell, and plasma cell genes (Figure 8D); this was also confirmed by IPA (Figure S3).

### MDTH Scores Correlate with Clinical RSV Disease Severity

**Figure 9:**
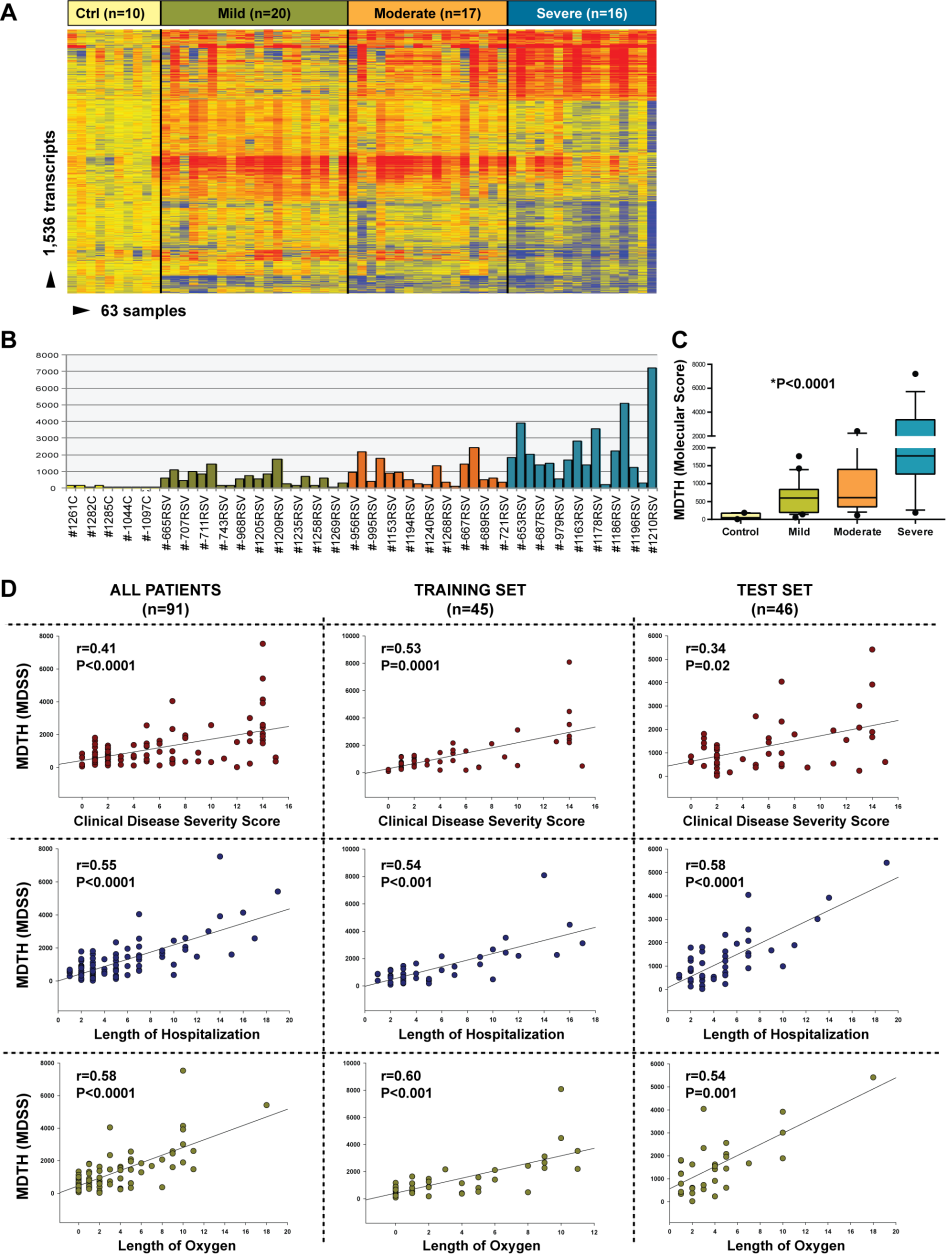
MDTH scores correlate with clinical disease severity in children with RSV LRTI. (A) Hierarchical clustering of 1,536 significantly differentially expressed transcripts (Kruskal-Wallis *p*<0.01, Benjamini-Hochberg multiple test correction) between 53 RSV patients classified as having mild (*n* = 20), moderate (*n* = 17), or severe (*n* = 16) RSV LRTI and ten healthy matched controls (Ctrl). (B) This gene list was used to calculate the MDTH score, or molecular disease severity score (MDSS). Each bar represents the MDTH score for a given sample (yellow bars represent the scores for healthy controls, green for mild RSV, orange for moderate RSV, and blue for severe RSV). (C) Children with severe RSV LRTI, and thus higher CDSSs, also had significantly greater MDTH scores (severe: median 1,769 [IQR 1,268–3,870] versus moderate: median 607 [IQR 350–1,396] and mild: median 596 [IQR 194–836]; Kruskal-Wallis test *p*<0.0001). (D) MDTH scores significantly correlated with CDSS, total length of hospitalization, and total duration of supplemental O_2_ in the overall RSV cohort (*n* = 91), and in the training set (*n* = 45) and test (*n* = 46) sets when calculated separately (Spearman's *r*).

We investigated whether blood transcriptional markers could help classifying RSV patients based on disease severity. Kruskal-Wallis tests (*p*<0.01) followed by multiple comparisons tests identified 1,536 significantly differentially expressed transcripts between children with mild, moderate, or severe disease and ten healthy controls. Hierarchical clustering of those genes demonstrated a higher proportion of underexpressed genes in children with severe RSV that gradually declined in patients with moderate and mild RSV disease, confirming the observations derived from the modular analysis (Figure 9A). Using those 1,536 transcripts, we calculated the MDTH scores. MDTH scores were higher in children with severe disease than in children with mild or moderate disease (*p*<0.001) (Figure 9B and 9C). In addition, the genomic MDTH score significantly correlated with the CDSS, the total length of hospitalization, and the duration of supplemental O_2_ in the overall RSV cohort (*n* = 91), and in the training (*n* = 45) and test sets (*n* = 41) when calculated separately (Figure 9D).

## Discussion

Despite the global impact of RSV, our understanding of the immunopathogenesis of this infection and how the different components of the host response relate to the clinical manifestations of the disease remains incomplete. Using an unbiased analytical strategy we found that the host systemic response to RSV (RSV biosignature) was robust, as it was validated in different patient populations from different countries and with diverse genetic backgrounds. We also found that the blood RSV biosignature was specific compared with those induced by influenza and HRV, and demonstrated that the immune dysregulation induced by RSV persisted beyond the acute disease, and it was greatly impaired in younger infants. Lastly, we identified a genomic score that significantly correlated with clinical indices of disease severity. We believe that this is the first study that provides a comprehensive analysis of whole blood gene expression profiles in infants with RSV LRTI, and that describes a significant association between gene expression profiles and clinical disease severity in the context of an acute viral LRTI.

To better understand the immunopathogenesis of RSV infection, investigators have performed gene expression analyses of cells in vitro [25]–[27] and in animal models [28]–[30]. Data in children are limited to two small studies. In the first study, gene expression profiles were characterized from cord blood samples in five infants at birth [31] and at the time of RSV hospitalization 3 mo later [32]. In a more recent study, whole blood gene profiles from 21 children with mild-to-moderate RSV bronchiolitis (median age of 6 mo) were characterized upon presentation to the emergency department [33]. Despite the paucity of data, and the known effect of the viral nonstructural proteins NS1/NS2 in counteracting interferon responses, activation of interferon-related genes was a common finding in those studies and was also found in our cohort of 135 infants with RSV infection. In the present study, we were also able to follow 21 infants with RSV infection and found an even greater activation of interferon-related genes in samples obtained at a follow-up visit 4–6 wk after the acute disease than in samples obtained during the acute disease from those same exact patients, suggesting that acute RSV infection partially inhibits interferon responses.

The systemic activation of common immune pathways in response to respiratory viral infections has been reported recently [34]. Zaas et al. experimentally challenged adult volunteers with RSV, influenza, and HRV and identified a blood gene expression signature that differentiated symptomatic infected individuals from uninfected individuals with 95% accuracy [35]. They found that interferon-related genes such as *RSAD2*, *LAMP3*, *IFI44L*, and *OAS1* were significantly differentially expressed in individuals with symptomatic RSV, influenza, or HRV infection. In agreement with those findings, the interferon signature and those specific genes (included in module M1.2) were overexpressed in our cohort of infants naturally infected with those viruses.

Studies have shown that influenza triggers a more robust immune response than RSV, with greater production of respiratory and systemic cytokines [36]–[38]. Our recent studies using human primary airway respiratory epithelial cells confirmed that RSV induced a less robust interferon and cytokine response than influenza at the mucosal level. Moreover, we found that the antiviral response of these cells to influenza and RSV correlated with the interferon signature derived from peripheral blood mononuclear cells isolated from patients with acute influenza or RSV bronchiolitis [27]. While peripheral blood responses may just reflect a leakage of immune cells or soluble factors originally activated by RSV in the respiratory tract, they could also be representative of a circulating subset of immune cells that do not contribute directly to the lung pathology but are involved in disease pathogenesis. In a study conducted in children with RSV or influenza LRTI who died, Welliver et al. showed the presence of an extensive antigen load with near absence of CD8^+^ cells and NK cells in the lungs. These findings partially support our results in whole blood, where we found greater underexpression of genes related to T cells and cytotoxic/NK cells in the most severe forms of RSV LRTI [38].

In the present study we also showed that systemic whole blood immune responses to RSV, influenza, and HRV were quantitatively and qualitatively different. RSV induced significant overexpression of neutrophil genes, which were less expressed, or even suppressed, in children with influenza or HRV infection. Although children with RSV LRTI had illnesses comparable with those of children with HRV or influenza in terms of the severity score or duration of hospitalization, RSV induced a more profound suppression of the adaptive immune response, as shown by the greater underexpression of T and B cell genes that mirrored the severity of the disease. It is remarkable that the suppression of B cell genes was still present when patients' samples were analyzed 1 mo after the acute infection. This profound suppression of B cells may explain, at least in part, the lack of protective antibody responses after acute RSV infection. Our results also indicate that RSV suppressed both the adaptive and innate responses more effectively in younger infants. These observations will require confirmation and further analysis using other approaches, as they may have implications for RSV vaccine development.

Our study has limitations. Study samples were obtained after laboratory confirmation of viral LRTI, a median of 2 d after hospital admission, which may in fact reflect a time point after the immune response and cytokine peak had occurred. In addition, the signatures were derived only from infants requiring hospitalization, which represents the tip of the iceberg of the disease spectrum caused by RSV. Nevertheless, it serves as a reference, and warrants future research including studies with earlier, sequential samples as well as samples from infants with milder RSV infection who are managed as outpatients. A great proportion of children in this study were of Hispanic background, which could limit the generalizability of the results. However, we validated the RSV signature in two independent cohorts of patients with different ethnic backgrounds enrolled in Finland and Ohio. Lastly, profiles were derived from peripheral whole blood and not from the respiratory mucosa, which may not necessarily reflect what is occurring in the lungs, the primary site of infection.

In conclusion, this study provides evidence of the profound systemic dysregulation of both the innate and adaptive immune response induced by RSV infection in children, and confirms the value of gene expression profiling as a practical and powerful strategy to objectively stratify children with acute RSV LRTI. Although this is a small study that will need to be replicated in a larger number of patients, we showed that the genomic scores derived from patients enrolled a median of 48 h after hospitalization correlated with total length of hospitalization and duration of supplemental oxygen. The practicality of using this molecular disease severity score for RSV bronchiolitis may have implications in the clinical setting. On one hand, it may help in triaging patients when they first present to the emergency department or pediatric office, but it could also be useful for monitoring clinical changes during the course of the disease, with the ultimate goal of predicting clinical outcomes. Indeed, there are novel and affordable PCR-based tools with faster turnaround time currently under development that will facilitate the application of transcriptional profiles in “real time” in the clinical setting. Ultimately, this study demonstrates that a large amount of microarray data can be translated into a biologically meaningful context that can be correlated with disease severity and applied in the relevant clinical setting.

## Supporting Information

Figure S1 **Hierarchical clustering of children with RSV, HRV, and influenza LRTI.** During six respiratory seasons (October 2006 to April 2011) we analyzed 241 whole blood microarray samples from 220 individuals less than 2 y of age hospitalized with LRTI: 135 from patients with RSV, 30 from patients with HRV, 16 from patients with influenza, 21 from follow-up RSV patients, and 39 from healthy matched controls (Table S1). A hierarchical clustering of all samples based on the quality control gene list (QC; 15,530 transcripts) is displayed. The dotted line separates the groups of children enrolled in Columbus, Ohio (controls *n* = 8; RSV cases *n* = 28), and Turku, Finland (controls *n* = 4; RSV cases *n* = 16). FU, follow-up.
(TIFF)

Figure S2 **Differences in host systemic responses depending on age in children with HRV and influenza A infection.** (A) Modular analysis in children with influenza A infection at 0–6 mo (*n* = 6) and 6–24 mo of age (*n* = 10) and matched healthy controls (*n* = 8 and *n* = 6, respectively) revealed significantly greater overexpression of neutrophils (M5.15) in younger infants, while genes related to interferon, inflammation, innate immunity, and plasma cells were significantly overexpressed in children 6–24 mo of age. (D) Modular analysis in children with HRV LRTI (<6 mo: *n* = 12 and 8 matched controls; 6–24 mo: *n* = 8 and 6 matched controls) revealed fewer differences in host responses according to age. (B and E) Horizontal bars illustrate the proportion of over- and underexpressed modules in infants <6 mo and children 6–24 mo of age in relation to the global influenza and HRV signature. (C and F). These differences are further illustrated in a spider graph representing the per-module median expression values of the significantly differentially expressed modules between the two age groups.
(TIFF)

Figure S3 **Canonical pathways according to RSV disease severity.** IPA canonical pathways for B cell and antigen-presenting cell signaling demonstrate greater suppression in children with severe RSV LRTI. Specific transcripts overexpressed (red) or suppressed (blue) were identified in those pathways.
(TIFF)

Table S1 **Study participant characteristics and allocation within analyses.** DS, disease severity; F/U, follow-up.
(DOCX)

Table S2 **Top ten over- and underexpressed genes in infants with RSV LRTI.**
(DOCX)

Table S3 **Expression of interferon-related genes in children with influenza, RSV, and HRV LRTI.** Interferon-related genes (*n* = 161) are comprised in modules M1.2, M3.4, and M5.12 (first column). Gene Probe names are displayed in the second column, and median expression values per gene/viral infection in the subsequent columns. Grey areas reflect no differences from healthy controls in expression values. Children with influenza displayed a stronger activation of interferon-related genes, 96.72% (155/161), compared with RSV, 76.39% (123/161), and HRV, 39.75% (64/161). Several type I interferon (IFIH1, IFIT1–5, STAT2, MX1) and type II interferon (IFI16, CXCL10 CCL8, GBP1–5, STAT1, SOCS1) genes were expressed only in influenza and RSV infection.
(DOCX)

## Abbreviations

CDSS: clinical disease severity score
HRV: human rhinovirus
IPA: Ingenuity Pathways Analysis
IQR: interquartile range
K-NN: *k*-nearest neighbor
LRTI: lower respiratory tract infection
MDTH: molecular distance to health
NK: natural killer
RSV: respiratory syncytial virus
