# Supplementary figures and images for: Whole Blood Gene Expression Profiles to Assess Pathogenesis and Disease Severity in Infants with Respiratory Syncytial Virus Infection
Source: PLoS Med. 2013 Nov 12;10(11):e1001549. doi: 10.1371/journal.pmed.1001549 (PMC3825655; doi:10.1371/journal.pmed.1001549)

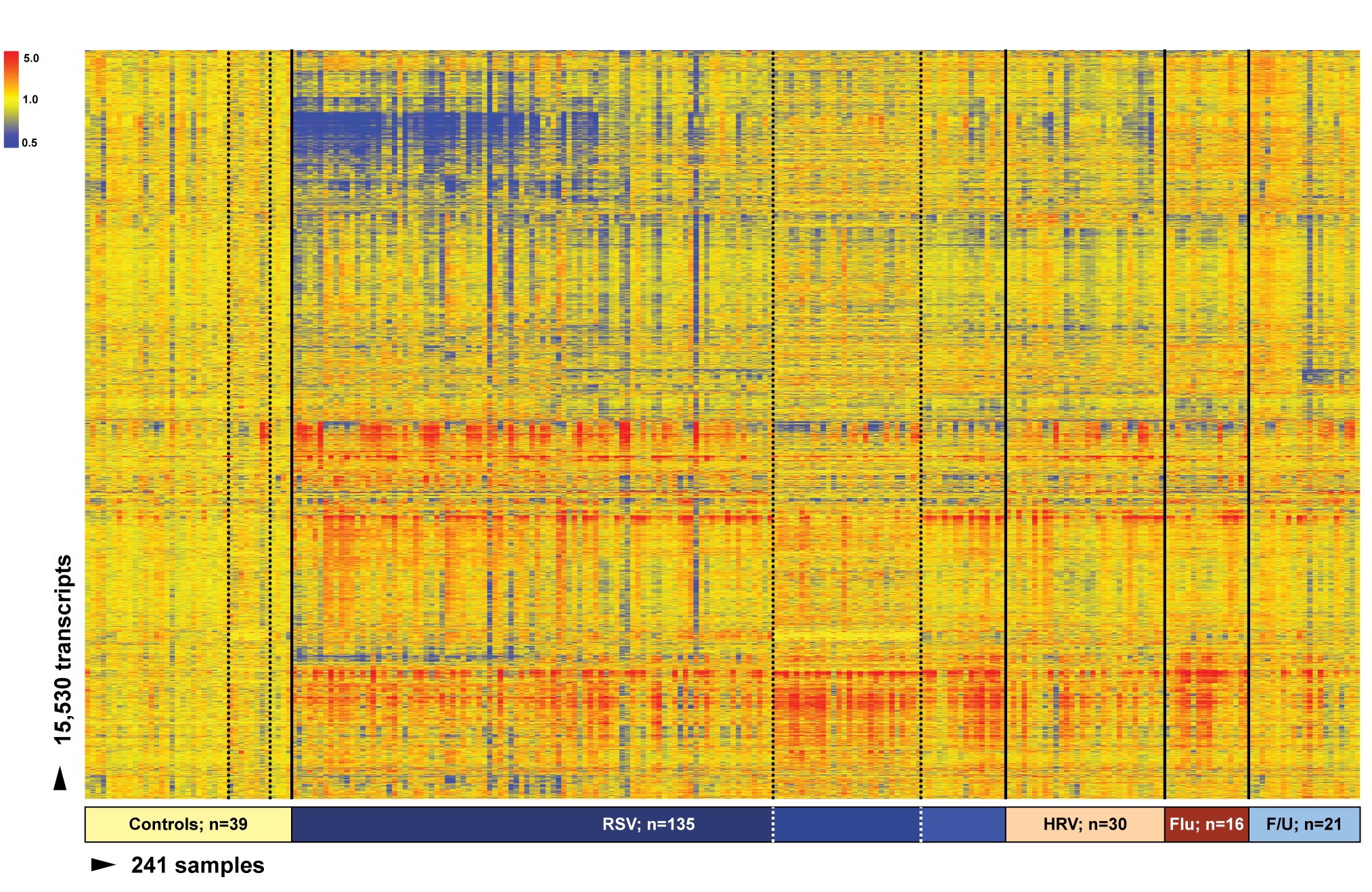

Supplement: Figure S1 — Hierarchical clustering of children with RSV, HRV, and influenza LRTI. During six respiratory seasons (October 2006 to April 2011) we analyzed 241 whole blood microarray samples from 220 individuals less than 2 y of age hospitalized with LRTI: 135 from patients with RSV, 30 from patients with HRV, 16 from patients with influenza, 21 from follow-up RSV patients, and 39 from healthy matched controls (Table S1). A hierarchical clustering of all samples based on the quality control gene list (QC; 15,530 transcripts) is displayed. The dotted line separates the groups of children enrolled in Columbus, Ohio (controls n = 8; RSV cases n = 28), and Turku, Finland (controls n = 4; RSV cases n = 16). FU, follow-up. (TIFF) [file pmed.1001549.s001.tif]

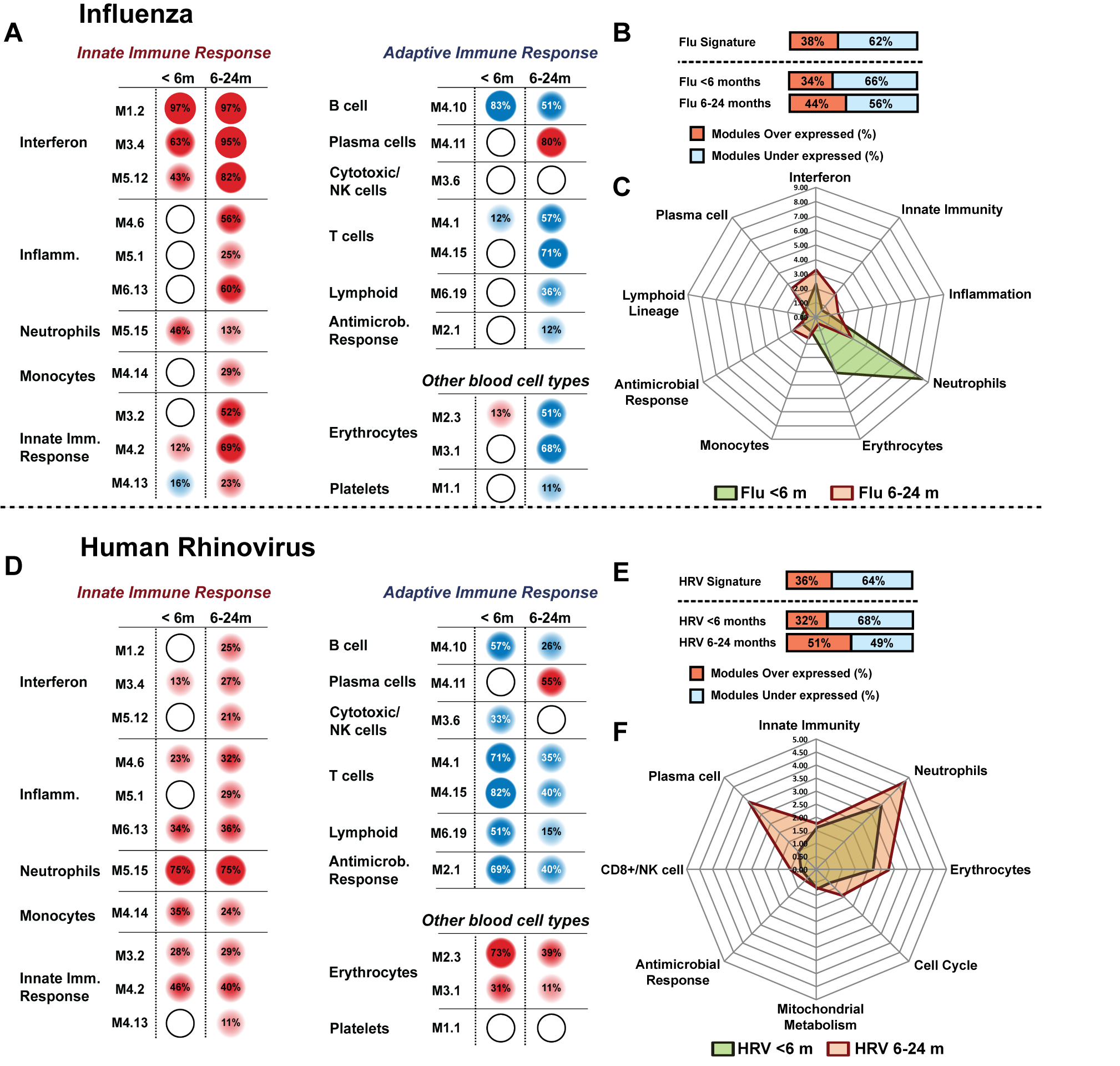

Supplement: Figure S2 — Differences in host systemic responses depending on age in children with HRV and influenza A infection. (A) Modular analysis in children with influenza A infection at 0–6 mo (n = 6) and 6–24 mo of age (n = 10) and matched healthy controls (n = 8 and n = 6, respectively) revealed significantly greater overexpression of neutrophils (M5.15) in younger infants, while genes related to interferon, inflammation, innate immunity, and plasma cells were significantly overexpressed in children 6–24 mo of age. (D) Modular analysis in children with HRV LRTI (<6 mo: n = 12 and 8 matched controls; 6–24 mo: n = 8 and 6 matched controls) revealed fewer differences in host responses according to age. (B and E) Horizontal bars illustrate the proportion of over- and underexpressed modules in infants <6 mo and children 6–24 mo of age in relation to the global influenza and HRV signature. (C and F). These differences are further illustrated in a spider graph representing the per-module median expression values of the significantly differentially expressed modules between the two age groups. (TIFF) [file pmed.1001549.s002.tif]

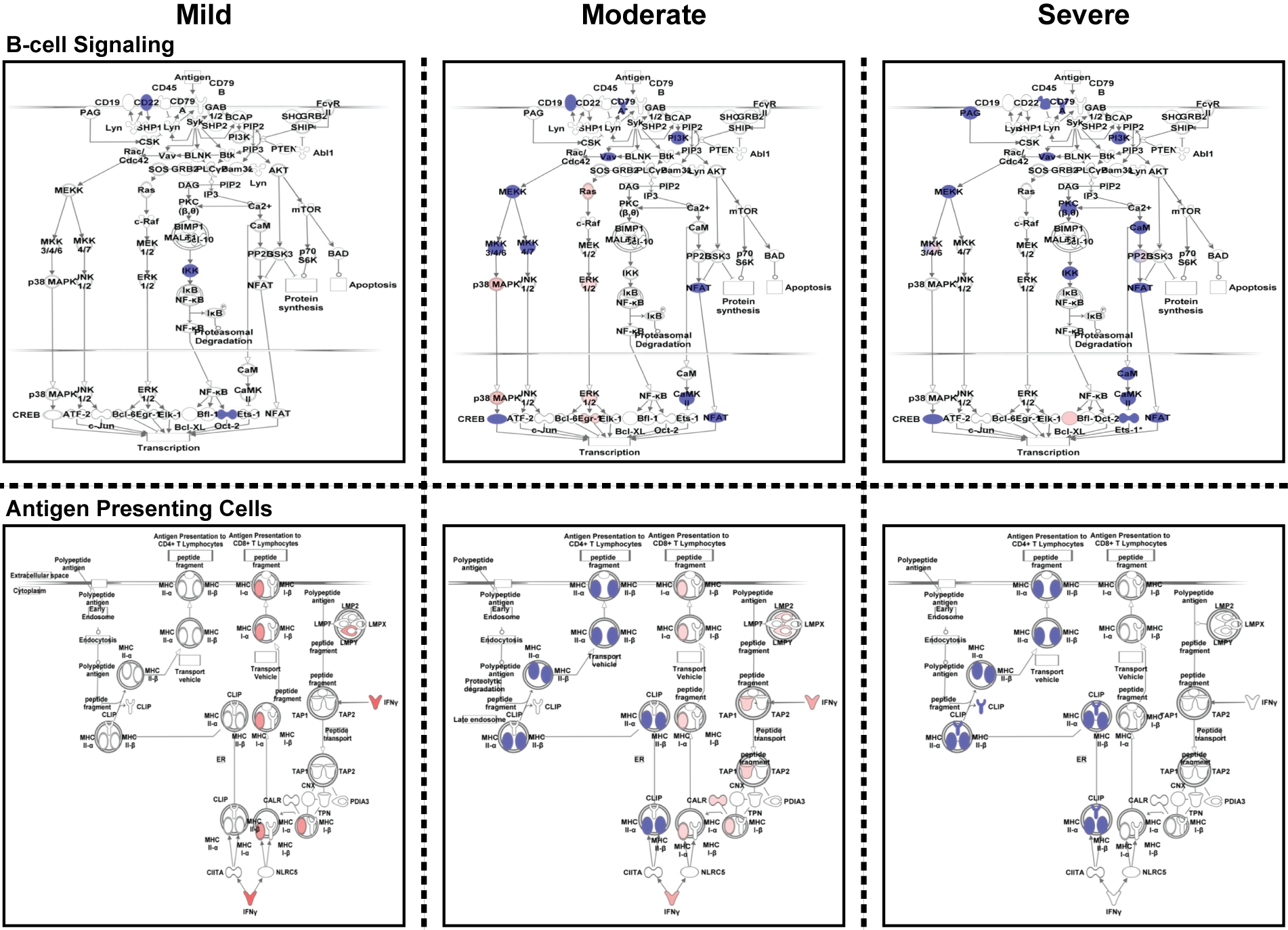

Supplement: Figure S3 — Canonical pathways according to RSV disease severity. IPA canonical pathways for B cell and antigen-presenting cell signaling demonstrate greater suppression in children with severe RSV LRTI. Specific transcripts overexpressed (red) or suppressed (blue) were identified in those pathways. (TIFF) [file pmed.1001549.s003.tif]
