# Supplementary material for: Whole Blood Gene Expression Profiles to Assess Pathogenesis and Disease Severity in Infants with Respiratory Syncytial Virus Infection
Source: PLoS Med. 2013 Nov 12;10(11):e1001549. doi: 10.1371/journal.pmed.1001549 (PMC3825655; doi:10.1371/journal.pmed.1001549)
Supplement: Table S1 — Study participant characteristics and allocation within analyses. DS, disease severity; F/U, follow-up. (DOCX) [file pmed.1001549.s004.docx]

**Table S1. Study Subject characteristics and allocation within analyses**

| Index # | **Age (months)** | **Sex** | **Race/Ethnicity** | **Condition** | **Analysis** |
| --- | --- | --- | --- | --- | --- |
| 1 | 1.9 | M | H | RSV | Training/Discrimination |
| 2 | 0.67 | F | H | RSV | Test/D.S/Discrimination |
| 3 | 2.33 | F | B | RSV | Training/D.S/Age/Discrimination |
| 4 | 8.03 | F | H | RSV | Training/Age/Discrimination |
| 5 | 5.53 | M | O | RSV | Training/Age/Discrimination |
| 6 | 1.9 | M | H | RSV | Training/Discrimination |
| 7 | 7.83 | F | H | RSV | Training/Age/Discrimination |
| 8 | 0.93 | M | W | RSV | Test/D.S/Discrimination |
| 9 | 1.03 | F | O | RSV | Training/Discrimination |
| 10 | 2.43 | M | H | RSV | Training/Age/Discrimination |
| 11 | 10.8 | F | H | RSV | Training/Age/Discrimination |
| 12 | 2.13 | M | H | RSV | Training/D.S/Age/Discrimination |
| 13 | 15.33 | F | H | RSV | Training/Age/Discrimination |
| 14 | 0.9 | M | H | RSV | Training/Discrimination |
| 15 | 12.57 | F | H | RSV | Training/Age/Discrimination |
| 16 | 3.1 | M | W | RSV | Training/D.S/Discrimination |
| 17 | 0.63 | M | B | RSV | Test/Discrimination |
| 18 | 4.3 | M | H | RSV | Training/Age/Discrimination |
| 19 | 2.13 | F | H | RSV | Training/D.S/Discrimination |
| 20 | 1.57 | F | H | RSV | Training/Discrimination |
| 21 | 2.03 | M | W | RSV | Training/Discrimination |
| 22 | 1.3 | F | W | RSV | Training/D.S/Discrimination |
| 23 | 2.27 | F | W | RSV | Training/D.S/Discrimination |
| 24 | 4.63 | F | W | RSV | Training/Age/Discrimination |
| 25 | 1.57 | F | H | RSV | Training/Discrimination |
| 26 | 3.6 | M | H | RSV | Test/Discrimination |
| 27 | 3.9 | F | B | RSV | Test/D.S/Age/Discrimination |
| 28 | 20.6 | M | H | RSV | Training/D.S/Age/Discrimination |
| 29 | 1.77 | M | W | RSV | Test/Discrimination |
| 30 | 2.3 | F | H | RSV | Test/D.S/Discrimination |
| 31 | 8.2 | F | H | RSV | Test/D.S/Age/Discrimination |
| 32 | 1.7 | M | H | RSV | Test/D.S/Discrimination |
| 33 | 18 | M | O | RSV | Training/D.S/Age/Discrimination |
| 34 | 0.43 | M | H | RSV | Test/D.S/Discrimination |
| 35 | 1.6 | F | W | RSV | Test/D.S/Discrimination |
| 36 | 1.87 | M | H | RSV | Test/D.S/Discrimination |
| 37 | 0.57 | F | H | RSV | Test/D.S/Discrimination |
| 38 | 0.63 | M | H | RSV | Test/D.S/Discrimination |
| 39 | 1.9 | F | H | RSV | Test/D.S/Discrimination |
| 40 | 4.43 | M | H | RSV | Test/D.S/Age/Discrimination |
| 41 | 10.13 | M | H | RSV | Training/D.S/Age/Discrimination |
| 42 | 0.67 | F | H | RSV | Test/D.S/Discrimination |
| 43 | 1.27 | M | H | RSV | Test/discrimination |
| 44 | 2.33 | M | W | RSV | Test/discrimination |
| 45 | 1.5 | F | H | RSV | Test/D.S/Age/Discrimination |
| 46 | 4.07 | M | H | RSV | Test/Age/Discrimination |
| 47 | 0.47 | M | H | RSV | Test/Discrimination |
| 48 | 1.33 | F | H | RSV | Test/Age/Discrimination |
| 49 | 0.7 | F | H | RSV | Test/D.S/Discrimination |
| 50 | 3.1 | M | H | RSV | Test/D.S/Age/Discrimination |
| 51 | 1.27 | F | H | RSV | Test/Age/Discrimination |
| 52 | 0.77 | M | H | RSV | Test/Discrimination |
| 53 | 1.3 | M | H | RSV | Training/D.S/Age/Discrimination |
| 54 | 1.57 | M | H | RSV | Training/D.S/Age/Discrimination |
| 55 | 6.37 | M | H | RSV | Training/D.S/Age/Discrimination |
| 56 | 1.17 | F | W | RSV | Training/D.S/Age/Discrimination |
| 57 | 5.43 | F | H | RSV | Training/D.S/Discrimination |
| 58 | 2.43 | F | W | RSV | Training/D.S/Age/Discrimination |
| 59 | 0.7 | F | W | RSV | Training/D.S/Discrimination |
| 60 | 2.73 | F | H | RSV | Training/D.S/Age/Discrimination |
| 61 | 1.63 | M | H | RSV | Training/D.S/Discrimination |
| 62 | 6.27 | F | B | RSV | Training/Age/Discrimination |
| 63 | 2.27 | M | H | RSV | Training/D.S/Age/Discrimination |
| 64 | 1.83 | M | H | RSV | Training/Discrimination |
| 65 | 1.3 | M | W | RSV | Training/D.S/Discrimination |
| 66 | 0.9 | M | H | RSV | Training/Discrimination |
| 67 | 0.97 | M | H | RSV | Training/D.S/Discrimination |
| 68 | 2.03 | F | H | RSV | Training/D.S/Discrimination |
| 69 | 0.6 | F | H | RSV | Test/D.S/Discrimination |
| 70 | 22.33 | M | H | RSV | Training/D.S/Age/Discrimination |
| 71 | 3.53 | M | H | RSV | Training/D.S/Age/Discrimination |
| 72 | 2.8 | M | H | RSV | Training/D.S/Discrimination |
| 73 | 1.13 | F | W | RSV | Test/Discrimination |
| 74 | 1.23 | M | W | RSV | Test/Discrimination |
| 75 | 1.87 | F | W | RSV | Test/Discrimination |
| 76 | 1.1 | M | H | RSV | Test/Discrimination |
| 77 | 2.5 | M | O | RSV | Test/D.S/Discrimination |
| 78 | 8.1 | M | H | RSV | Test/Age/Discrimination |
| 79 | 1.27 | M | W | RSV | Test/D.S/Discrimination |
| 80 | 0.57 | F | H | RSV | Test/Discrimination |
| 81 | 2.67 | F | W | RSV | Test/D.S/Discrimination |
| 82 | 2.93 | F | B | RSV | Test/D.S/Discrimination |
| 83 | 1.77 | F | W | RSV | Test/D.S/Discrimination |
| 84 | 16.77 | F | H | RSV | Training/Age/Discrimination |
| 85 | 2.87 | F | H | RSV | Test/Discrimination |
| 86 | 11.4 | M | H | RSV | Test/D.S/Age/Discrimination |
| 87 | 2.93 | M | H | RSV | Test/D.S/Discrimination |
| 88 | 0.37 | F | O | RSV | Test/D.S/Discrimination |
| 89 | 8.03 | F | B | RSV | Test/Age/Discrimination |
| 90 | 18.4 | F | O | RSV | Test/Age/Discrimination |
| 91 | 0.53 | F | H | RSV | Test/D.S/Discrimination |
| 92 | 4.3 | F | W | RSV | Validation A |
| 93 | 20.27 | F | W | RSV | Validation A |
| 94 | 6.87 | F | W | RSV | Validation A |
| 95 | 5.6 | M | W | RSV | Validation A |
| 96 | 12.37 | F | W | RSV | Validation A |
| 97 | 4.13 | M | W | RSV | Validation A |
| 98 | 14.97 | F | W | RSV | Validation A |
| 99 | 3.33 | F | W | RSV | Validation A |
| 100 | 3.5 | M | W | RSV | Validation A |
| 101 | 10.53 | M | W | RSV | Validation A |
| 102 | 3.3 | F | W | RSV | Validation A |
| 103 | 7.67 | M | W | RSV | Validation A |
| 104 | 14 | F | W | RSV | Validation A |
| 105 | 3.97 | F | W | RSV | Validation A |
| 106 | 12.83 | M | W | RSV | Validation A |
| 107 | 6.07 | M | W | RSV | Validation A |
| 108 | 0.37 | M | W | RSV | Validation B |
| 109 | 4.40 | M | W | RSV | Validation B |
| 110 | 1.00 | F | W | RSV | Validation B |
| 111 | 4.00 | M | W | RSV | Validation B |
| 112 | 6.93 | F | W | RSV | Validation B |
| 113 | 4.60 | M | W | RSV | Validation B |
| 114 | 5.00 | F | W | RSV | Validation B |
| 115 | 2.53 | M | W | RSV | Validation B |
| 116 | 2.80 | M | B | RSV | Validation B |
| 117 | 3.90 | M | W | RSV | Validation B |
| 118 | 2.93 | M | B | RSV | Validation B |
| 119 | 0.53 | M | W | RSV | Validation B |
| 120 | 1.33 | F | H | RSV | Validation B |
| 121 | 5.33 | F | W | RSV | Validation B |
| 122 | 2.73 | M | O | RSV | Validation B |
| 123 | 1.60 | F | W | RSV | Validation B |
| 124 | 12.77 | F | B | RSV | Validation B |
| 125 | 0.87 | M | W | RSV | Validation B |
| 126 | 3.03 | F | W | RSV | Validation B |
| 127 | 2.90 | M | W | RSV | Validation B |
| 128 | 2.27 | F | W | RSV | Validation B |
| 129 | 4.07 | M | W | RSV | Validation B |
| 130 | 3.33 | F | W | RSV | Validation B |
| 131 | 1.50 | F | W | RSV | Validation B |
| 132 | 2.00 | M | W | RSV | Validation B |
| 133 | 1.70 | M | W | RSV | Validation B |
| 134 | 8.43 | M | H | RSV | Validation B |
| 135 | 3.73 | M | W | RSV | Validation B |
| 136 | 12.4 | M | W | Healthy control | Validation A |
| 137 | 11 | F | W | Healthy control | Validation A |
| 138 | 7.9 | M | W | Healthy control | Validation A |
| 139 | 6.4 | F | W | Healthy control | Validation A |
| 140 | 1.20 | M | W | Healthy control | Validation B |
| 141 | 11.83 | M | W | Healthy control | Validation B |
| 142 | 12.27 | M | W | Healthy control | Validation B |
| 143 | 13.60 | M | B | Healthy control | Validation B |
| 144 | 8.83 | M | W | Healthy control | Validation B |
| 145 | 1.00 | M | W | Healthy control | Validation B |
| 146 | 1.60 | F | W | Healthy control | Validation B |
| 147 | 1.93 | M | W | Healthy control | Validation B |
| 148 | 18.43 | M | H | Healthy control | Age/Test |
| 149 | 10.5 | M | B | Healthy control | Age/Test |
| 150 | 11.77 | M | H | Healthy control | Age/Test |
| 151 | 17.47 | F | H | Healthy control | Age/Test |
| 152 | 8.63 | F | H | Healthy control | Age/Test |
| 153 | 5.67 | F | H | Healthy control | Training/Age |
| 154 | 4.2 | M | W | Healthy control | Training/D.S/Age |
| 155 | 6.8 | F | W | Healthy control | Training/D.S |
| 156 | 7.2 | F | B | Healthy control | Training/D.S |
| 157 | 9.27 | M | B | Healthy control | Training/D.S/Age |
| 158 | 3.93 | F | H | Healthy control | Training/D.S/Age |
| 159 | 2.27 | M | H | Healthy control | Training/D.S/Age |
| 160 | 4.1 | M | H | Healthy control | Training/D.S/Age |
| 161 | 6.13 | F | O | Healthy control | Training/D.S/Age |
| 162 | 6.17 | F | H | Healthy control | Training/D.S |
| 163 | 6.7 | F | H | Healthy control | Training/D.S |
| 164 | 3.97 | F | H | Healthy control | Training/D.S/Age |
| 165 | 1.07 | F | W | Healthy control | Training/D.S/Age |
| 166 | 4.13 | F | H | Healthy control | Training/Age |
| 167 | 12.23 | M | B | Healthy control | Age/Test |
| 168 | 9.8 | F | W | Healthy control | Test/D.S/Age |
| 169 | 15.5 | F | B | Healthy control | Test/HRV Normalization |
| 170 | 2.93 | F | H | Healthy control | Test/HRV Normalization |
| 171 | 2.23 | M | H | Healthy control | Test/HRV Normalization |
| 172 | 0.96 | M | H | Healthy control | Test/HRV Normalization |
| 173 | 0.3 | F | B | Healthy control | Test/HRV Normalization |
| 174 | 2.67 | M | H | Healthy control | Test/D.S/Age |
| 175 | 1.17 | M | W | HRV | Discrimination |
| 176 | 1.83 | M | H | HRV | Discrimination |
| 177 | 1.2 | M | H | HRV | Discrimination |
| 178 | 1.6 | M | B | HRV | Discrimination |
| 179 | 18.37 | F | W | HRV | Discrimination |
| 180 | 10.73 | F | O | HRV | Discrimination |
| 181 | 0.4 | M | H | HRV | Discrimination |
| 182 | 15.2 | F | H | HRV | Discrimination |
| 183 | 0.67 | F | H | HRV | Discrimination |
| 184 | 1.67 | F | W | HRV | Discrimination |
| 185 | 9.87 | F | H | HRV | Discrimination |
| 186 | 7.6 | M | B | HRV | Discrimination |
| 187 | 0.6 | M | W | HRV | Discrimination |
| 188 | 0.67 | M | H | HRV | Discrimination |
| 189 | 2.77 | F | B | HRV | Discrimination |
| 190 | 0.73 | M | B | HRV | Discrimination |
| 191 | 10.03 | F | H | HRV | Discrimination |
| 192 | 2.77 | M | B | HRV | Discrimination |
| 193 | 1.7 | M | H | HRV | Discrimination |
| 194 | 2.2 | M | O | HRV | Discrimination |
| 195 | 2.6 | M | H | HRV | Discrimination |
| 196 | 5.07 | F | H | HRV | Discrimination |
| 197 | 13.43 | M | H | HRV | Discrimination |
| 198 | 1.63 | M | W | HRV | Discrimination |
| 199 | 2 | F | H | HRV | Discrimination |
| 200 | 3.6 | M | H | HRV | Discrimination |
| 201 | 3.67 | F | H | HRV | Discrimination |
| 202 | 2.2 | M | H | HRV | Discrimination |
| 203 | 6.5 | M | H | HRV | Discrimination |
| 204 | 0.7 | M | H | HRV | Discrimination |
| 205 | 2.87 | M | H | Influenza A | Discrimination |
| 206 | 6.33 | M | B | Influenza A | Discrimination |
| 207 | 6.33 | M | B | Influenza A | Discrimination |
| 208 | 4.43 | F | H | Influenza A | Discrimination |
| 209 | 1.2 | M | H | Influenza A | Discrimination |
| 210 | 0.97 | F | H | Influenza A | Discrimination |
| 211 | 9.93 | M | W | Influenza A | Discrimination |
| 212 | 9.3 | F | H | Influenza A | Discrimination |
| 213 | 6.4 | F | W | Influenza A | Discrimination |
| 214 | 1.4 | F | B | Influenza A | Discrimination |
| 215 | 6.97 | M | H | Influenza A | Discrimination |
| 216 | 2.33 | F | O | Influenza A | Discrimination |
| 217 | 14.1 | M | H | Influenza A | Discrimination |
| 218 | 14.87 | F | B | Influenza A | Discrimination |
| 219 | 18.83 | F | W | Influenza A | Discrimination |
| 220 | 7.1 | F | H | Influenza A | Discrimination |
| 221 | 1.6 | F | W | RSV F/U | Follow up analysis |
| 222 | 2.4 | F | H | RSV F/U | Follow up analysis |
| 223 | 7.53 | M | H | RSV F/U | Follow up analysis |
| 224 | 2.56 | M | H | RSV F/U | Follow up analysis |
| 225 | 3.23 | M | H | RSV F/U | Follow up analysis |
| 226 | 2.5 | M | H | RSV F/U | Follow up analysis |
| 227 | 2.5 | M | H | RSV F/U | Follow up analysis |
| 228 | 3.73 | M | O | RSV F/U | Follow up analysis |
| 229 | 1.6 | F | H | RSV F/U | Follow up analysis |
| 230 | 17.86 | F | H | RSV F/U | Follow up analysis |
| 231 | 9.13 | F | B | RSV F/U | Follow up analysis |
| 232 | 19.83 | F | O | RSV F/U | Follow up analysis |
| 233 | 6.2 | M | H | RSV F/U | Follow up analysis |
| 234 | 5.53 | M | H | RSV F/U | Follow up analysis |
| 235 | 3.36 | M | H | RSV F/U | Follow up analysis |
| 236 | 3.36 | M | H | RSV F/U | Follow up analysis |
| 237 | 1.96 | F | O | RSV F/U | Follow up analysis |
| 238 | 9.13 | F | H | RSV F/U | Follow up analysis |
| 239 | 6.76 | M | O | RSV F/U | Follow up analysis |
| 240 | 9.86 | F | H | RSV F/U | Follow up analysis |
| 241 | 1.7 | F | H | RSV F/U | Follow up analysis |
