# Supplementary material for: Whole Blood Gene Expression Profiles to Assess Pathogenesis and Disease Severity in Infants with Respiratory Syncytial Virus Infection
Source: PLoS Med. 2013 Nov 12;10(11):e1001549. doi: 10.1371/journal.pmed.1001549 (PMC3825655; doi:10.1371/journal.pmed.1001549)
Supplement: Table S2 — Top ten over- and underexpressed genes in infants with RSV LRTI. (DOCX) [file pmed.1001549.s005.docx]

**Table S2. Top 10 Over and Underexpressed Genes in Infants with RSV LRTI**
