# Supplementary material for: Whole Blood Gene Expression Profiles to Assess Pathogenesis and Disease Severity in Infants with Respiratory Syncytial Virus Infection
Source: PLoS Med. 2013 Nov 12;10(11):e1001549. doi: 10.1371/journal.pmed.1001549 (PMC3825655; doi:10.1371/journal.pmed.1001549)
Supplement: Table S3 — Expression of interferon-related genes in children with influenza, RSV, and HRV LRTI. Interferon-related genes (n = 161) are comprised in modules M1.2, M3.4, and M5.12 (first column). Gene Probe names are displayed in the second column, and median expression values per gene/viral infection in the subsequent columns. Grey areas reflect no differences from healthy controls in expression values. Children with influenza displayed a stronger activation of interferon-related genes, 96.72% (155/161), compared with RSV, 76.39% (123/161), and HRV, 39.75% (64/161). Several type I interferon (IFIH1, IFIT1–5, STAT2, MX1) and type II interferon (IFI16, CXCL10 CCL8, GBP1–5, STAT1, SOCS1) genes were expressed only in influenza and RSV infection. (DOCX) [file pmed.1001549.s006.docx]

**Table S3. Expression of Interferon related genes in children with influenza, RSV and HRV LRTI.**
